# Integrative Multi-Omics Reveals TSPAN13 as a Candidate Epithelial–Mesenchymal Transition-Associated Regulator in Prostate Cancer

**DOI:** 10.3390/biomedicines14102241

**Published:** 2026-10-03

**Authors:** Shuo Lu, Houhe Li, Zixiao Zhang, Dafu Yu, Yuming Qiao, Junyu Ouyang, Shaowen Liu, Hua Wang, Ke Li

**Affiliations:** 1 Department of Urology, The Third Affiliated Hospital, Sun Yat-sen University, Guangzhou 510630, China; lush33@mail2.sysu.edu.cn (S.L.); lihh88@mail2.sysu.edu.cn (H.L.); yudf3@mail2.sysu.edu.cn (D.Y.); liushw36@mail2.sysu.edu.cn (S.L.); 2 Department of Urology, Zhaoqing Hospital, The Third Affiliated Hospital, Sun Yat-sen University, Zhaoqing 526071, China; zhangzx29@mail2.sysu.edu.cn (Z.Z.); m15247705405@163.com (Y.Q.); ouyangjy1996@163.com (J.O.)

**Keywords:** prostate cancer, epithelial–mesenchymal transition, single-cell RNA sequencing, machine learning, molecular dynamics

## Abstract

**Background**: Prostate cancer (PCa) is a malignancy with high metastatic potential and poor prognosis, underscoring the urgent need for effective biomarkers and therapeutic targets to improve clinical management. **Methods**: An integrative multi-omics analysis combined with machine learning algorithms was performed to identify key diagnostic biomarkers. Candidate genes were validated through extensive functional assays in vitro, including proliferation, migration, invasion, and epithelial–mesenchymal transition (EMT) analyses. The tumor-promoting effect of TSPAN13 was further assessed in vivo using xenograft mouse models. Immune cell infiltration patterns were analyzed to explore associations with the tumor microenvironment. **Results**: An exploratory diagnostic model centered on TSPAN13 was developed. Functional depletion of TSPAN13 significantly suppressed PCa cell proliferation, migration, invasion, and EMT in vitro. These anti-tumor effects were recapitulated in vivo, where TSPAN13 knockdown markedly inhibited tumor growth without significant change in body weight. Furthermore, TSPAN13 expression was correlated with distinct alterations in the immune cell composition within the tumor microenvironment. **Conclusions**: This study identifies TSPAN13 as a candidate EMT-associated regulator in PCa with potential diagnostic and therapeutic relevance. Our findings provide novel insights into PCa pathogenesis and highlight TSPAN13 as a potential candidate for preclinical exploration, offering potential for strategies targeting epithelial malignancy and modulating the immune microenvironment.

## 1. Introduction

Prostate cancer (PCa) is the most common cancer diagnosis in men, accounting for 30% of male cancers in 2025, and is the second leading cancer death in men behind lung cancer [1]. As a malignant tumor, PCa represents an increasingly prominent threat to human health and stands as a major challenge in the field of global public health [2]. While about 75% of PCa patients are diagnosed with the localized form and experience a nearly 100% 5-year survival rate, the outlook is far more severe for the roughly 10% diagnosed with metastatic disease, who have a 5-year survival rate of just 37% [3]. Although earlier detection and incremental therapeutic gains have been made, metastatic castration-resistant PCa remains a lethal disease. This underscores the need to investigate non-androgen pathways driving tumor survival and therapeutic escape [4]. Normally in metazoans, prostate epithelial cells form a tightly packed layer anchored to the basal membrane by junctional proteins. In development, however, these cuboidal cells dramatically alter their identity through epithelial–mesenchymal transition (EMT), acquiring a more elongated and spindle-like form characteristic of mesenchymal cells [5]. Critically, this same developmental mechanism is co-opted during tumorigenesis. The malignant transformation of PCa follows a multi-step progression, starting with prostatic intraepithelial neoplasia, advancing to localized disease, then to locally invasive and advanced adenocarcinoma, and ultimately culminating in metastatic PCa. Throughout this process, the interaction between tumor cells and the surrounding muscle cells is identified as a significant risk factor [6]. In cancer progression, the EMT program is hijacked by malignant cells, endowing them with invasive and migratory properties. This transformation enables them to degrade the basement membrane and initiate metastasis [7]. The molecular profile of EMT reflects a dramatic shift in cellular identity. This process involves the downregulation of key epithelial markers (e.g., E-cadherin, occludin, ZO-1) and is concurrently replaced by the expression of hallmark mesenchymal markers (e.g., vimentin, N-cadherin, fibronectin) [8]. Upon transitioning to a mesenchymal state, cells acquire heightened capacities for migration and invasion, gain resistance to apoptosis, and develop an enhanced ability to remodel the extracellular matrix [9]. Tumors often harbor hybrid cell populations that co-express both epithelial and mesenchymal markers, a plastic state which enhances metastatic potential. This reprogramming involves the enzymatic dissolution of basement membrane attachments. Coupled with phenotypic alterations—such as the reorganization of the actin cytoskeleton—these changes collectively equip cells for migration and metastasis [10,11]. Identifying and validating EMT-associated regulator could lead to actionable biology that may be translated into therapeutic interventions.

Tetraspanins (TSPANs) are a widely expressed superfamily of transmembrane proteins (20–50 kDa), first identified in 1990. They are involved in key physiological and pathological processes and are noted for their evolutionarily conserved sequences across diverse species [12]. Functionally, TSPANs operate by forming dimers or heterodimers to interact with partner proteins such as integrins and adhesion molecules. In humans, the 33-member family plays key regulatory roles through these interactions, governing processes including cell proliferation, migration, apoptosis, autophagy, and immune responses [13]. TSPAN13 is a 204-amino-acid membrane protein located on chromosome 7p21.1 [14] and is significantly dysregulated in several malignancies, including breast cancer [15], papillary thyroid cancer [16] and acute myeloid leukemia [17]. However, the specific functions and regulatory mechanisms of TSPAN13 in PCa remain incompletely understood. Addressing this gap, our study employed an integrated multi-data approach coupled with robust machine learning algorithms to systematically identify and prioritize candidate EMT-associated regulators that are central to PCa pathogenesis. Furthermore, we extended our investigation beyond cell-autonomous effects to explore the critical relationship between TSPAN13 expression levels and the composition of the tumor immune microenvironment. This analysis revealed compelling associations suggesting TSPAN13 status may influence immune cell infiltration and activity, thereby revealing potential implications for the development or efficacy of immunotherapeutic strategies in PCa subsets.

Collectively, our findings provide a deeper, more integrated understanding of the molecular landscape of prostate epithelial cells in PCa. We not only elucidate the functional significance of TSPAN13 but also nominate it as a candidate biomarker with diagnostic potential, and as rational targets for novel therapeutic approaches aimed at mitigating epithelial cells-associated PCa progression.

## 2. Materials and Methods

### 2.1. Data Acquisition

The single-cell RNA sequencing (scRNA-seq) data analyzed in this study were obtained from the public dataset GSE176031, which includes 7 radical prostatectomy paired normal and 8 radical prostatectomy paired tumor. Cell quality control involved the removal of low-quality cells based on two key metrics: cells with fewer than 50 detected genes (indicating potential droplet failures or empty droplets) and cells with a mitochondrial gene percentage greater than 15% (indicating compromised cellular integrity). We performed all data preprocessing, filtering, and subsequent analyses using the Seurat (v4.4.1) R package [18,19]. Briefly, gene expression profiles were re-normalized, and principal component analysis (PCA) was conducted based on 1500 highly variable genes. The optimal dimensionality for clustering was selected by evaluating Elbow and JackStraw plots. Cell clusters were visualized in two dimensions using uniform manifold approximation and projection (UMAP). Differentially expressed genes across clusters were identified with the FindAllMarkers function (Wilcoxon test; thresholds: adj. *p*-value < 0.05, |log FC| > 1). Finally, cell types were annotated and visualized with the SingleR package (v2.2.0) [20]. Two publicly available microarray datasets (GSE60329, GSE71781) were downloaded from the Gene Expression Omnibus (GEO) database. Probe-level expression matrices were converted to gene-level expression profiles using platform-specific annotation files. Standardized preprocessing procedures were implemented, including quantile normalization, inter-platform variation harmonization, and batch effect correction via established algorithm (Figure S1A,B). PCA plots were generated to validate the effectiveness of batch correction by comparing data distributions before and after adjustment (Figure S1C,D).

### 2.2. Cell–Cell Communication Analysis

Intercellular communication networks were interrogated with the CellChat package [21] for a systematic analysis of ligand–receptor interactions across cell types in the PCa microenvironment. The CellChat object was created from the normalized RNA expression matrix and cell metadata, using the human Secreted Signaling database (CellChatDB.human) as the reference for ligand–receptor pairs. After preprocessing and identifying overexpressed genes within each cell type, communication probabilities were computed based on ligand–receptor co-expression patterns, with interactions involving fewer than 10 cells filtered out. The resulting communication networks were aggregated and visualized at multiple levels: circle plots depicted the overall number and strength of interactions between cell types; hierarchical plots organized signaling pathways by their information flow; chord diagrams illustrated specific ligand–receptor pairs; and bubble plots provided a comprehensive overview of signaling pathway activity across all cell type pairs.

### 2.3. High-Dimensional Weighted Gene Co-Expression Network Analysis (hdWGCNA)

We performed hdWGCNA on the epithelial cell subset to identify gene modules associated with PCa states. The analysis was conducted using the hdWGCNA R package (v0.4.06) [22]. After filtering for genes expressed in at least 5% of cells, we constructed metacells grouped by cell type and sample origin (benign/tumor) using harmony reduction and k = 25 nearest neighbors to mitigate single-cell data sparsity. A signed co-expression network was built following SCTransform normalization, employing a soft-thresholding power of β = 7 to achieve scale-free topology. Gene modules were identified through hierarchical clustering and dynamic tree cutting of the topological overlap matrix. Subsequently, we computed harmonized module eigengenes, quantified module membership (kME) for all genes, and identified hub genes. The resulting modules were characterized through various visualizations, including feature plots, correlation networks, and a gene UMAP based on hub genes.

### 2.4. Functional Enrichment Analysis of Key Epithelial Modules

To elucidate the biological functions of the key epithelial cell-derived gene modules, Gene Ontology (GO), Kyoto Encyclopedia of Genes and Genomes (KEGG) pathway enrichment and Disease Ontology (DO) enrichment analysis were performed. Hub genes (top 25 genes based on module membership, kME) from the all modules (Epithelial cells—M1, Epithelial cells—M2, Epithelial cells—M3 and Epithelial cells—M4) were extracted from the hdWGCNA results. Functional enrichment was conducted using the clusterProfiler R package (v4.12.6), with statistical significance set at an adjusted *p*-value < 0.05. The analysis aimed to identify significantly overrepresented biological processes, molecular functions, cellular components, signaling pathways and capture of all potential disease association signals associated with these pivotal epithelial cell modules in the PCa microenvironment.

### 2.5. Mendelian Randomization (MR) Study Design and Data Sources

The genetic instruments for exposures were sourced from the eQTLGen consortium, comprising cis-eQTL summary data for multiple genes in European populations. The outcome data for PCa were obtained from the GWAS catalog (ieu-b-85, https://opengwas.io/datasets/, accessed on 12 August 2025), which includes 79,148 cases and 61,106 controls. All datasets were formatted consistently to ensure uniformity, with essential information including single-nucleotide polymorphisms (SNPs), effect alleles, effect sizes, standard errors, and corresponding *p*-values extracted for analysis.

### 2.6. MR Selection of Instrumental Variables (IVs)

Instrumental variables for gene expression exposures were selected under stringent criteria to ensure robust genetic association inference. A genome-wide significance threshold of *p* < 5 × 10^−8^ was applied for IV selection. Linkage disequilibrium (LD) clustering was performed with an R^2^ < 0.001 and a clumping distance of 10,000 kb to ensure genetic independence among variants [23]. The strength of each genetic instrument was quantitatively assessed using the F-statistic, calculated from the proportion of variance explained (R^2^) and sample size. Only instruments with F-statistics > 10 were retained for subsequent MR analysis to minimize potential bias from weak instruments [24].

To measure the association between the exposure and the outcome, we first estimate the percentage of variability accounted for by the exposure, represented as R^2^. This value is obtained using the effect size and allele frequency of the exposure variable, as outlined in the following formula [25]:

$$R^{2}=\frac{2\cdot \beta_{\mathrm{exposure}}^{2}\cdot {\mathrm{eaf}}_{\mathrm{exposure}}\cdot (1-{\mathrm{eaf}}_{\mathrm{exposure}})}{2\cdot \beta_{\mathrm{exposure}}^{2}\cdot {\mathrm{eaf}}_{\mathrm{exposure}}\cdot \left(1-{\mathrm{eaf}}_{\mathrm{exposure}}\right)+2\cdot {\mathrm{se}}_{\mathrm{exposure}}^{2}\cdot n_{\mathrm{exposure}}\cdot {\mathrm{eaf}}_{\mathrm{exposure}}\cdot \left(1-{\mathrm{eaf}}_{\mathrm{exposure}}\right)}$$

Subsequently, to evaluate the statistical significance of the model, we calculate the F statistic by relating the proportion of variance explained to the sample size, as expressed in the following formula:

$$F=\frac{R^{2}\cdot \left(n_{\mathrm{exposure}}-2\right)}{1-R^{2}}$$

*β_exposure_*: This denotes the estimated effect size of the exposure variable, equivalent to its regression coefficient.

*eaf_exposure_*: This refers to the frequency of the effect allele for the exposure variable within the studied population.

*se_exposure_*: This indicates the standard error associated with the estimated effect (β) of the exposure variable.

*n_exposure_*: This corresponds to the total sample size used in the analysis for the exposure.

### 2.7. MR Analysis for Biomarker Discovery

The PCa GWAS summary statistics in VCF format (ieu-b-85.vcf.gz) were converted to TwoSampleMR-compatible format using the gwasglue package. For the MR analysis, we utilized locally stored eQTL data as exposures and the converted PCa GWAS data as the outcome. The exposure dataset comprised instrument variables that passed previous quality control (F-statistic > 10). Data harmonization was performed to align effect alleles between exposure and outcome datasets, ensuring consistency in effect direction. Only SNPs present in both exposure and outcome datasets were retained for subsequent analysis.

We implemented a systematic MR pipeline for each eQTL gene exposure. Analyses were restricted to genes with at least three valid instrumental variables after harmonization. The MR analysis incorporated multiple methods including inverse variance weighted (IVW), MR-Egger, weighted median, and simple mode. For each gene, we generated odds ratios with 95% confidence intervals and performed comprehensive sensitivity analyses: (1) MR-Egger intercept test for directional pleiotropy (*p* > 0.05 considered acceptable); (2) Cochran’s Q test for heterogeneity; (3) leave-one-out analysis to identify influential variants; (4) funnel plots for visual assessment of asymmetry. Significant associations were defined as those with IVW *p*-value < 0.05 and consistent effect directions across all MR methods. For all significant gene-outcome associations, we generated four diagnostic plots: scatter plots depicting the SNP-outcome associations against SNP-exposure associations, forest plots showing individual SNP effects, funnel plots for asymmetry assessment, and leave-one-out plots evaluating the influence of individual variants.

### 2.8. Construction of the Diagnostic Nomogram, Calibration, and Decision Curve Analysis

A diagnostic nomogram was constructed using the rms R package (v6.8-2) based on the merged and normalized dataset. The outcome was sample type (Control vs. Tumor), and the candidate feature gene was used as predictors in a logistic regression model. Calibration was assessed by bootstrap resampling. Decision curve analysis was performed for TSPAN13 with 95% confidence intervals.

### 2.9. Immune Microenvironment Profiling

We employed the CIBERSORT algorithm to quantify the abundance of 22 immune cell populations in tissue transcriptomes derived from bulk RNA-seq data [26]. The analysis utilized the CIBERSORTx platform’s R implementation, incorporating the LM22 signature matrix for cell type identification. Computational workflows were executed in R (v4.4.1) with dependency on ‘e1071’ (v1.7-16), ‘preprocessCore’ (v1.66.0), and ‘parallel’ (v4.4.1) libraries. Prior to deconvolution, transcriptomic profiles underwent quantile normalization via the preprocessCore package (v1.66.0). Visualization strategies included compositional bar charts with color-coded sample annotations to display cell type distributions. Correlation matrices between immune subsets were generated using Spearman’s rho and presented as dendrogram-clustered heatmaps through the corrplot package (v0.95) (hclust method). Differential abundance testing between experimental groups was performed using non-parametric Wilcoxon tests, with results illustrated via ggplot2/ggpubr-generated boxplots. Statistical thresholds followed * *p* < 0.05, ** *p* < 0.01, and *** *p* < 0.001 conventions. Key parameters for reproducibility included 1000 bootstrap iterations in CIBERSORT for *p*-value calibration for cell type inclusion. All analytical procedures were implemented in R environment, ensuring robust quantification of tumor–immune interactions.

### 2.10. Gene Set Variation Analysis (GSVA)

GSVA was performed using the ssGSEA algorithm to evaluate the enrichment scores of Hallmark gene sets in the transcriptomic data. The expression matrix was preprocessed and normalized prior to analysis. Enrichment scores were calculated using the GSVA package (v1.52.3) [27] with default parameters, and the resulting scores were normalized to a 0–1 scale for subsequent analysis. Differential pathway activity between experimental groups was assessed using boxplot visualizations with statistical significance determined by Wilcoxon tests. Furthermore, Spearman correlation analysis was conducted to examine the relationships between selected feature genes and Hallmark pathway activities, with results visualized through a correlation heatmap.

### 2.11. Molecular Docking Analysis

We implemented an integrated bioinformatics workflow combining network analysis and molecular modeling. Drug–gene interaction data were retrieved from the DSigDB database for comprehensive enrichment analysis. To investigate the molecular interactions between TSPAN13 and its putative binding partners (Table S1), we performed detailed binding mode and affinity analysis. The crystal structure of the candidate EMT-associated regulator protein was obtained from the RCSB Protein Data Bank. Using PyMOL (v2.5.2), the protein structure was refined by removing water molecules and native ligands. The refined protein was subsequently imported into AutoDock Tools 1.5.7 for preprocessing, including hydrogenation, charge assignment, and optimization of non-polar hydrogen bonds. Molecular docking was executed using AutoDock Vina via command-line interface with meticulously defined grid box parameters and genetic algorithm settings. Finally, PyMOL was employed to visualize the docking results, providing insights into the binding interactions between TSPAN13 and putative binding partners.

### 2.12. Molecular Dynamics (MD) Simulation

Molecular dynamics simulations were conducted using GROMACS 2022.2 [28]. The topology and parameters for the small-molecule ligand were prepared by calculating its restrained electrostatic potential charges and optimizing its geometry using Gaussian 16, followed by parameter assignment with the CHARMM General Force Field. The protein–ligand complex was solvated in a TIP3P water box, neutralized with Na^+^ ions, and modeled using the CHARMM36 force field. The simulation protocol consisted of energy minimization via the steepest descent algorithm, followed by stepwise equilibration—first under an NVT ensemble for 4 ns at 300 K (Berendsen thermostat, τ_t_ = 0.1 ps), then under an NPT ensemble for 4 ns at 1 bar (Berendsen barostat, τ_p_ = 2.0 ps). Finally, a production run was conducted for 100 ns with a 2 fs timestep under constant temperature (300 K, V-rescale thermostat) and pressure (1 bar, Parrinello–Rahman barostat). Trajectory analysis included the calculation of root-mean-square deviation (RMSD) and fluctuation (RMSF) for the protein backbone, radius of gyration (Rg), and solvent-accessible surface area (SAS). Protein–ligand interactions were further characterized by monitoring the minimum intermolecular distance, the number of atomic contacts within 0.6 nm, and the decomposed non-bonded interaction energy. Collective motions were examined via principal component analysis (PCA) of the Cα-atom covariance matrix; the resulting eigenvalues and projection along the first principal component were analyzed. The Gibbs free energy landscape was constructed as a function of the first two principal components. Additionally, the binding free energy was estimated using the MM/PBSA approach on frames extracted from the stabilized trajectory.

### 2.13. Cell Line and Culture

PCa cell lines (PC3, DU145) and the normal prostate cell line RWPE1 were procured from the Cell Bank of the Chinese Academy of Sciences (Shanghai, China), with authenticated short tandem repeat profiles and mycoplasma-free certification. Cells were cultured in RPMI-1640 (Gibco, Grand Island, NY, USA) supplemented with 10% fetal bovine serum (FBS, Gibco, USA) under standard conditions (37 °C, 5% CO_2_). Subculturing employed 0.25% trypsin-EDTA (Gibco, USA), with a 1:3 split ratio. Cryopreservation utilized complete medium with 10% DMSO for long-term storage in liquid nitrogen.

### 2.14. Lentiviral Transfection and Quantitative Real-Time Polymerase Chain Reaction (qRT-PCR)

Lentiviral particles were produced in PCa cell lines PC3 and DU145 cells using a third-generation packaging system. Briefly, cells were seeded in 10 cm dishes at 70–80% confluency. For each dish, a transfection mixture was prepared by combining 10 μg of pLKO.1-shTSPAN13 (target plasmid), 6.4 μg of pSPAX2 (packaging plasmid), and 3.6 μg of pMD2.G (envelope plasmid) with 40 μL of P3000 reagent in 1.5 mL of Opti-MEM. Separately, 41 μL of Lipofectamine 3000 was diluted in 1.5 mL of Opti-MEM. The diluted Lipofectamine was then mixed with the plasmid-P3000 complex and incubated for 20 min at room temperature. The mixture was added dropwise to the cells, followed by incubation for 6–8 h before replacing the medium with fresh growth medium. Viral supernatants were harvested 72 h post-transfection, clarified by centrifugation, and stored at −80 °C. For infection, target cells were incubated with viral supernatant supplemented with 8 μg/mL polybrene. Stable cell lines were selected using 5 μg/mL puromycin for 3 days, with daily medium renewal. For qRT-PCR validation, total RNA extracted using TRIzol (Thermo Fisher Scientific, Waltham, MA, USA) was reverse-transcribed with RevertAid (Vazyme, Nanjing, China). SYBR Green-based qPCR (Accurate Biology, Changsha, China) on Roche LightCycler 480 used GAPDH-normalized primers: TSPAN13 (F: TCAACCTGCTTTACACCTTGG, R: CGGAGACTGGAAATCAGCCC) and GAPDH (F: GAGAAGGCTGGGGCTCATTT, R: GTCAAAGGTGGAGGAGTGGG). Protein validation via Western blot followed standard protocols.

### 2.15. Western Blot (WB)

Total proteins were extracted from cellular using RIPA lysis buffer (Thermo Fisher Scientific, USA) containing 1% protease inhibitor cocktail to ensure protein integrity. Lysates were centrifuged at 12,000× *g* rpm for 10 min at 4 °C, and supernatants were collected. Protein concentrations were determined using a BCA assay kit (Beyotime, Shanghai, China). Equal amounts of protein (30 μg/lane) were separated by 10% sodium dodecyl sulfate–polyacrylamide gel electrophoresis at constant voltage (120 V) until the dye front reached the bottom of the gel. Proteins were subsequently transferred onto polyvinylidene fluoride (PVDF) membranes (Millipore, Burlington, MA, USA) using a wet electroblotting system (Bio-Rad, Hercules, CA, USA) at 80 V for 40 min. Membranes were blocked with 5% non-fat dry milk in PBST (phosphate-buffered saline, 0.1% Tween-20) buffer for 1 h at room temperature with gentle shaking. After blocking, membranes were incubated overnight at 4 °C with primary antibodies: rabbit polyclonal TSPAN13 (1:1000 dilution, Abclonal, Wuhan, China) and rabbit monoclonal β-actin (1:2000 dilution, Proteintech, Wuhan, China). Following three 10 min washes in PBST, membranes were incubated with HRP-conjugated goat anti-rabbit secondary antibodies (1:5000 dilution, Abclonal, Wuhan, China) for 1 h at room temperature. After additional washing steps, protein bands were developed using enhanced chemiluminescence (ECL) reagent (Vazyme, Nanjing, China) and visualized with a Tanon Imaging System. All Western blot experiments were performed in three independent biological replicates (*n* = 3), and representative blots are shown.

### 2.16. Cell-Counting Kit 8 (CCK-8) Assay

PCa cell lines were allocated into negative control (sh-NC) group and TSPAN13-targeting shRNA group. Cells were seeded at an optimized density of 1 × 10^3^ cells/well (96-well plate format) in 100 μL complete medium and incubated overnight at 37 °C under 5% CO_2_ to ensure adherence. At experimental initiation (0 h), culture medium was gently aspirated, and cells were washed twice with 200 μL phosphate-buffered saline (PBS, pH 7.4) to remove non-adherent debris. Fresh RPMI-1640 medium (100 μL/well) supplemented with 10% FBS was then added to maintain physiological conditions. CCK-8 reagent (10 μL/well; Dojindo, Kumamoto, Japan) was introduced at 0, 24, 48, 72 and 96 h post-treatment. Following 2 h incubation at 37 °C, absorbance was measured at 450 nm using a BioTek Synergy H1MF (BioTek Instruments, Winooski, VT, USA). All experiments were performed in technical triplicates, with data normalized to 0 h baseline values for proliferation analysis.

### 2.17. Transwell Migration and Invasion Assay

Cell migration and invasion capacities were evaluated using 24-well Transwell inserts (8.0 μm pore size; Corning, Corning, NY, USA). For invasion assays, inserts were pre-coated with 50 μL Matrigel (1:8 dilution in serum-free RPMI-1640; BD Biosciences, Milpitas, CA, USA) and incubated at 37 °C for 2 h to form a reconstituted basement membrane. Uncoated inserts served as migration controls. Prior to assays, cells were serum-starved for 12 h in RPMI-1640. Suspensions were prepared at 1 × 10^5^ cells/mL in serum-free medium, and 200 μL aliquots were added to upper chambers. Lower chambers contained 600 μL RPMI-1640 with 10% FBS as a chemoattractant. Following 24 h incubation at 37 °C, non-migratory/non-invasive cells on the upper membrane surface were removed using cotton swabs. Migrated/invaded cells on the lower surface were fixed with 4% paraformaldehyde (Servicebio, Wuhan, China) for 20 min, stained with 0.1% crystal violet (Beyotime, Shanghai, China) for 10 min, and rinsed gently with PBS. Stained cells were imaged at 100× magnification using a Nikon microscope (Ti2-U, Nikon, Tokyo, Japan).

### 2.18. Scratch Wound Healing Assay

Scratch wound healing assays were conducted to evaluate migratory capacity in shRNA-transfected PCa cells (sh-NC and sh-TSPAN13 groups). Cells were seeded at 1 × 10^5^ cells/well in 6-well plates and cultured for 24 h at 37 °C under 5% CO_2_ to achieve ~90% confluence. Using a sterile 10 μL pipette tip, uniform linear wounds were created perpendicular to the cell monolayer. Debris was removed by two gentle washes with PBS, followed by incubation in RPMI-1640 medium containing 1% FBS to minimize proliferative effects. Wound-closure progression was monitored through Nikon microscope (Ti2-U, Japan) at 0 h and 12 h post-wounding.

### 2.19. Colony Formation Assay

Cells were seeded in 6-well plates at optimized densities (1000 cells/well) and cultured for 7 days under standard conditions to permit colony development. Following incubation, cellular monolayers were gently washed with PBS and fixed with 4% paraformaldehyde (Servicebio, China) for 20 min at room temperature. Fixed colonies were stained with 0.1% crystal violet solution (Beyotime, China) for 10 min with gentle rocking to ensure uniform dye penetration. Stained colonies were digitized using a Nikon microscope Imaging System (Ti2-U, Japan), with images captured in TIFF format for subsequent analysis.

### 2.20. EDU Proliferation Assay

Cell proliferation was assessed using the EdU (5-ethynyl-2′-deoxyuridine) incorporation assay (Beyotime). PC3 and DU145 cells with stable TSPAN13 knockdown (sh-TSPAN13-1, sh-TSPAN13-2) and negative control (sh-NC) were labeled with EdU for 2 h, followed by fixation, permeabilization, and Click-iT reaction with Azide 594. Nuclei were counterstained with DAPI. EdU-positive cells were visualized and quantified under a fluorescence microscope.

### 2.21. TdT-Mediated dUTP Nick-End Labeling (TUNEL) Assay

Apoptosis was evaluated using the TdT-mediated dUTP nick-end labeling (TUNEL) assay. Cells were fixed, permeabilized, and incubated with TUNEL detection solution at 37 °C for 60 min in the dark. Nuclei were counterstained with DAPI. TUNEL-positive (green) and DAPI-positive (blue) cells were imaged using fluorescence microscopy.

### 2.22. In Silico Gene Knockout Analysis Using scTenifoldKnk

In silico knockout analysis of TSPAN13 was performed using the scTenifoldKnk (v1.0.2) [29] framework to assess regulatory perturbations at the single-cell level. A preprocessed Seurat object containing PCa scRNA-seq data was used as input. Highly variable genes were identified using Seurat, and the top 5000 variable genes together with TSPAN13 were used for gene regulatory network construction. Gene regulatory networks were inferred from randomly subsampled cells using principal component regression, and multiple subnetworks were integrated to obtain a robust consensus network. The knockout was simulated by removing regulatory connections associated with TSPAN13, and perturbation effects were quantified by comparing network-derived gene regulation scores between knockout and wild-type networks. During network construction, cells with mitochondrial gene proportions greater than 10% or expressing fewer than 1000 genes were excluded. A total of 10 subnetworks were generated, each constructed from 500 randomly sampled cells, in accordance with recommended parameter settings. Differential regulatory perturbations were assessed, and significantly affected genes were identified based on adjusted *p* < 0.05. Fold changes derived from this analysis reflect the magnitude of regulatory network perturbation rather than direct transcriptional up- or downregulation.

### 2.23. In Vivo Study

All animal experiments were conducted using five-week-old male BALB/c nude mice (specific pathogen-free, SPF grade). Prior to the experiment, the mice were acclimatized to the SPF animal housing facility for 1 week to adapt to the environmental conditions. The housing environment was strictly controlled with a temperature of 22–26 °C, relative humidity of 50–60%, and a 12 h light/dark cycle. Sterilized food and filtered water were provided ad libitum throughout the acclimatization and experimental periods to ensure animal welfare.

After acclimatization, the mice were randomly assigned into experimental groups (*n* = 5 per group) using a random number table to minimize individual differences and ensure group homogeneity. The stable DU145 human PCa cell line was used for tumor xenograft establishment. The cell concentration was adjusted to 1 × 10^6^ cells/mL using a hemocytometer, and the cell suspension was freshly prepared by mixing with Matrigel (Corning, USA) at a final volume of 200 µL per injection (containing 1 × 10^6^ DU145 cells and 40% Matrigel). Matrigel was kept on ice throughout the preparation process to prevent premature solidification. Following tumor cell inoculation, the mice were monitored daily for general health status, including activity level, food intake, drinking behavior, and the presence of abnormal signs (e.g., weight loss, skin lesions, or distress). Tumor growth was monitored every four days by measuring the longest diameter (length) and the shortest diameter (width) of the subcutaneous tumors with a digital vernier caliper. Tumor volume (V) was calculated using the standard formula: V = (length × width^2^)/2. The body weight of each mouse was also recorded every four days to assess the systemic effects of tumor growth and to ensure animal welfare. All animal experimental procedures were reviewed and approved by the Animal Care and Use Committee of Sun Yat-sen University (Approval No. SYSU-IACUC-2026-B0002).

### 2.24. Statistical Analysis

Data processing, statistical analysis, and plotting were conducted using R software (v4.4.1). The Wilcoxon rank-sum test or t-test was used for continuous variable comparisons. Pearson’s correlation coefficient was used to assess the correlation between two continuous variables. *p* < 0.05 was considered statistically significant (* *p* < 0.05, ** *p* < 0.01, *** *p* < 0.001, ns: not significant).

## 3. Results

### 3.1. Single-Cell Transcriptional Landscape from PCa Patients

**Figure 1:**
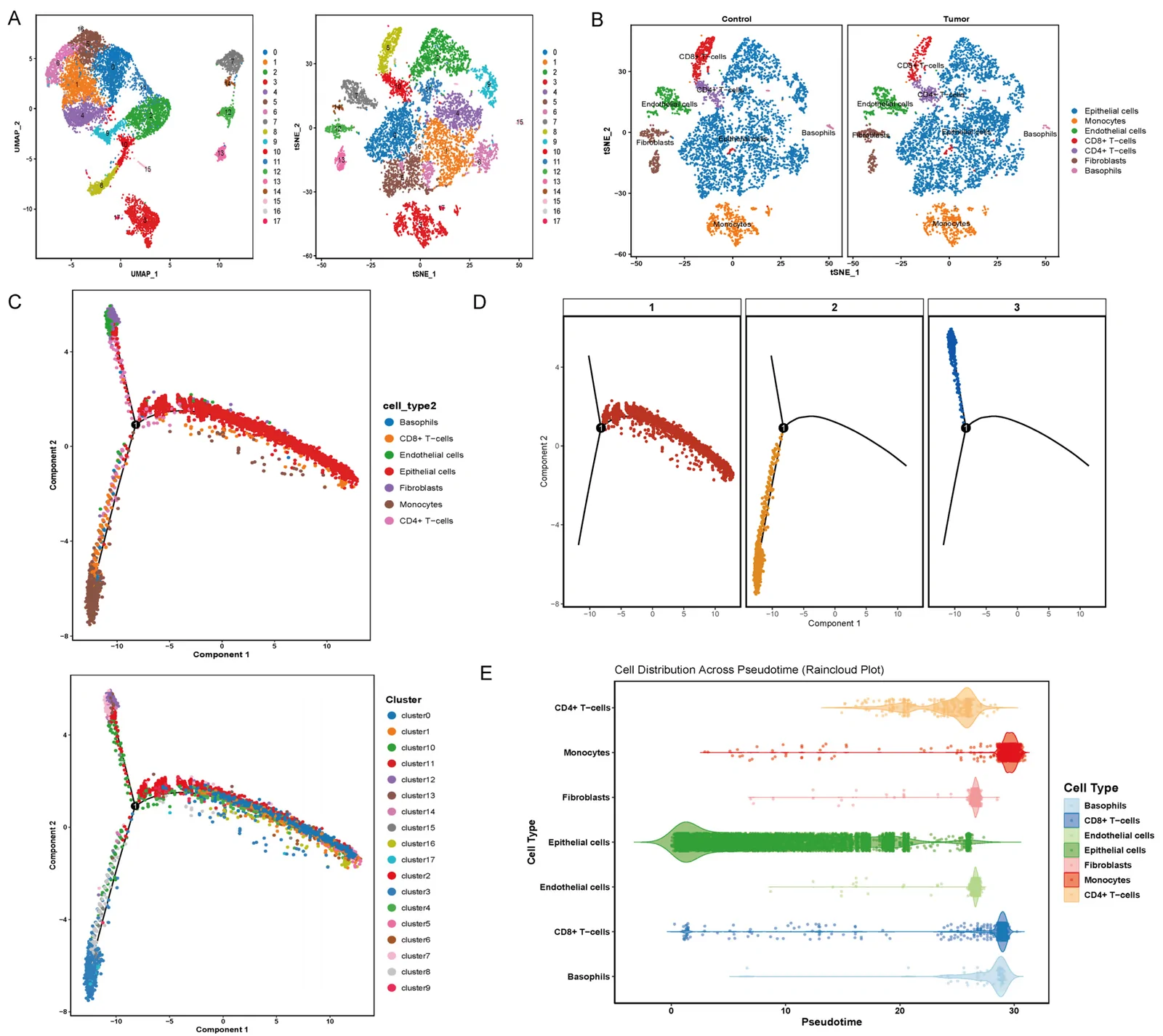
Identification of differentially expressed genes (DEGs) in PCa. (**A**) Unbiased clustering separates cells into 18 distinct populations, each marked by a unique colour UMAP and t-SNE visualisation showing cell clusters between the tumor group and the control group. (**B**) The 7 cell types were identified by marker genes, showing cell types between the tumor group and the control group. (**C**) Pseudotime trajectory analysis predicting the evolution of tumor cells across the 18 identified clusters and 7 cell types. The number 1 indicates branch 1 in the pseudotime trajectory analysis. (**D**) Pseudotime trajectory analysis predicting the evolution of tumor cells across the 18 identified clusters and within three unique tumoral cell states. (**E**) Raincloud Plot analysis predicting the evolution of tumor cells within 7 cell types.

To explore patient heterogeneity in PCa, we analyzed scRNA-seq data. After standardization, rigorous quality control, and preprocessing (Figure S2A–H), a high-quality dataset was obtained. Unsupervised clustering revealed 18 distinct cellular clusters. The differential expression of the top 10 marker genes across these clusters is displayed in a heatmap and t-SNE plot. Finally, manual annotation identified seven major cell populations: Epithelial cells, Monocytes, Endothelial cells, CD8^+^ T cells, CD4^+^ T cells, Fibroblasts, and Basophils (Figure 1A,B). Pseudotime trajectory analysis revealed a developmental path that originated from a common source, bifurcating into two distinct lineages (Figure 1C,D), which culminated in the formation of three unique tumoral cell states. The distribution of cells along the pseudotime continuum, as visualized by the raincloud plot (Figure 1E), further supports this branching model, showing distinct cellular densities corresponding to the divergent paths and endpoint states. Heatmap displaying the differential expression of the top 10 marker genes across the 18 identified clusters (Figure S3). Dynamic expression patterns of representative differentially expressed genes (DEGs) within each cluster, displayed through line graphs. Heatmap representation of representative DEGs across cell clusters, showcasing distinct gene expression profiles. GO enrichment analysis of representative DEGs, highlighting functional annotations specific to each cluster (Figure S4).

### 3.2. CellChat Inference of Cell–Cell Communication Between Multiple Cell Populations in PCa

**Figure 2:**
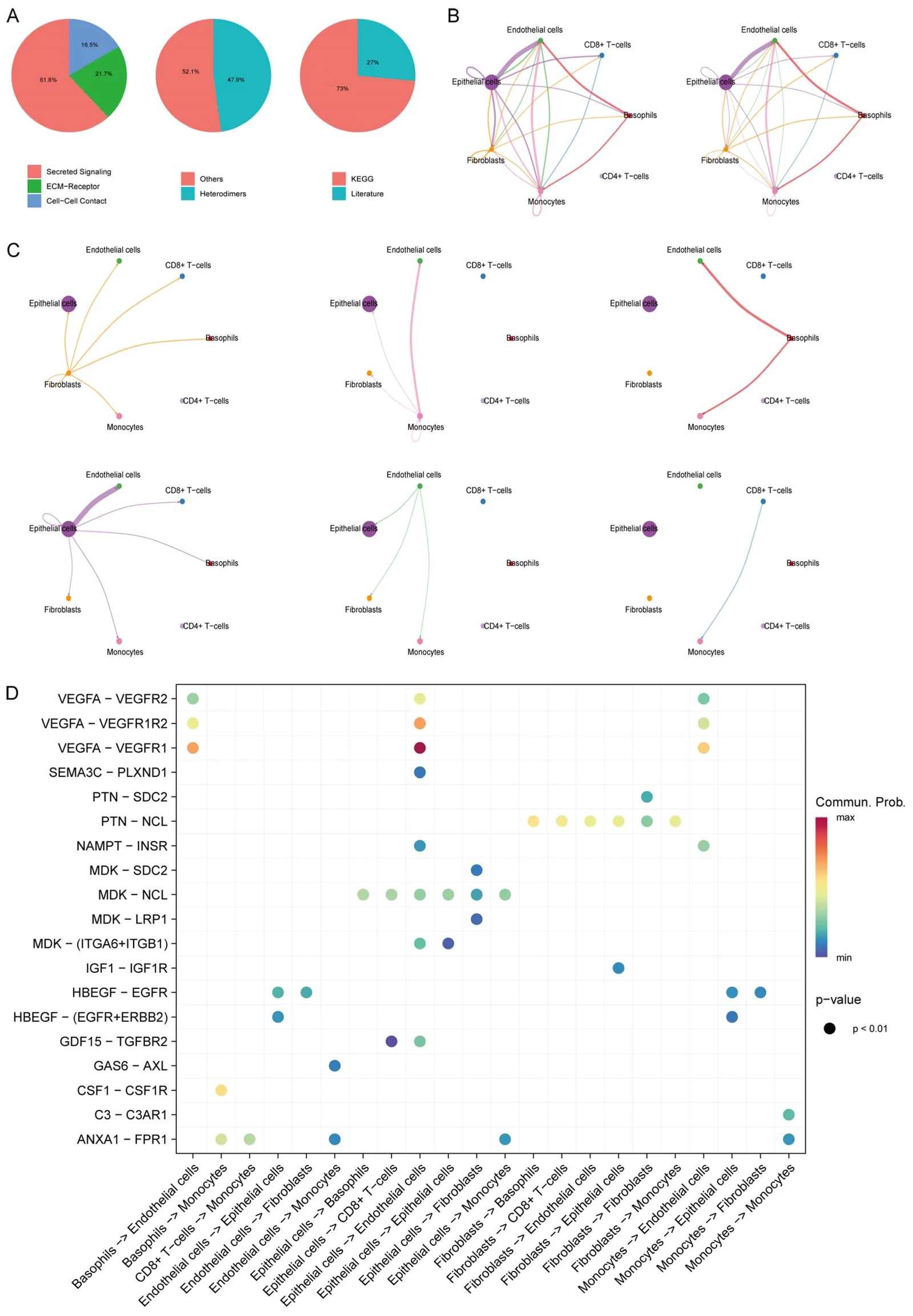
Schematic overview of the major categories of cell–cell interactions. (**A**) Based on their distinct spatial mechanisms—ranging from long-range diffusion of soluble ligands to direct membrane contact and cell-ECM adhesion—these three fundamental categories orchestrate cell–cell communication to regulate development, homeostasis and tissue dynamics. (**B**) The number and weights/strength of interactions in the cell–cell communication network. (**C**) Weight of classical inflammatory activation pathways. (**D**) Visualization of the signal intensity of ligand interaction.

We first systematically categorized the ligand–receptor interactions available in the CellChatDB database. As shown in Figure 2, the interactions in this database are primarily classified into three major categories: (1) Secreted Signaling refers to classical communication where ligands are secreted as soluble molecules into the extracellular matrix and subsequently bind to their cognate receptors, representing the primary mode of long-range intercellular communication. (2) ECM–Receptor Interactions reflect interactions between cells and extracellular matrix components, involving processes such as cell adhesion and migration. (3) Cell–Cell Contact Includes direct interactions between membrane-bound ligands and receptors on adjacent cell surfaces, mediating short-range contact-dependent cellular communication (Figure 2A). Analysis of global signaling cross-talk quantified the overall cell–cell interaction landscape, presenting the number and strength of interactions in circle plots where line thickness denotes intensity (Figure 2B). This revealed extensive and complex intercellular communication networks involving all cell types in PCa (Figure 2C). Interrogation of ligand–receptor pairs further identified crucial mediators of this cross-talk (Figure 2D). These results elucidate the specific cellular interactions and signaling pathways that drive tumor progression, highlighting the complexity of the immune network within the PCa microenvironment.

### 3.3. Co-Expression Network Analysis

**Figure 3:**
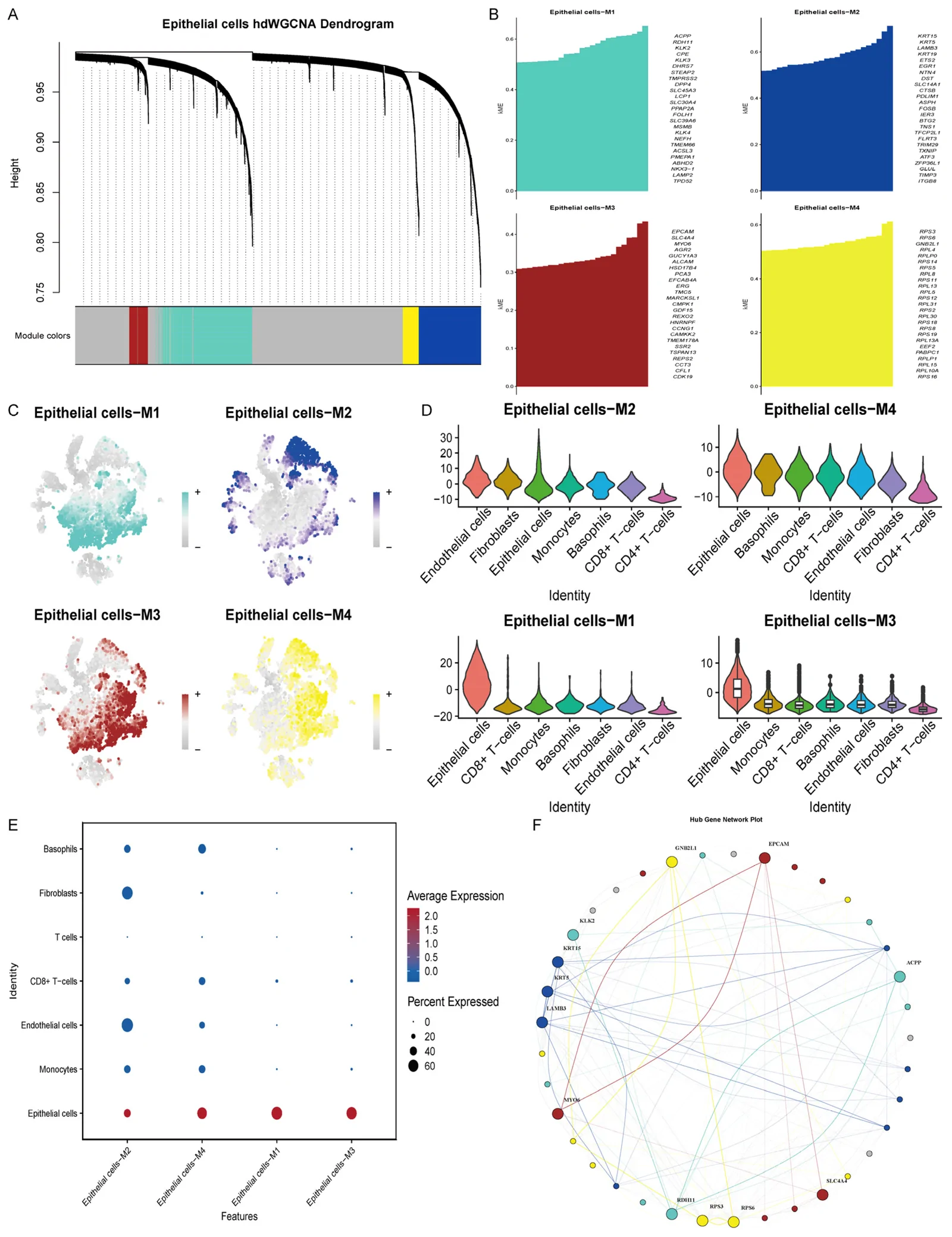
hdWGCNA Identifies Key Hub Modules Associated with Epithelial cells. (**A**) Hierarchical clustering dendrogram of genes based on topological overlap, with branches annotated by co-expression modules. (**B**) Distribution of eigengene-based connectivity (kME) for genes in each module, highlighting module hub genes. (**C**) scRNA-seq t-SNE visualization of cells colored by harmonized module eigengene (hME) scores, illustrating module activity across cells. (**D**) Violin plots of module expression scores across cell types, with overlaid boxplots summarizing central tendency and variability. (**E**) Dot plot showing correlations between module eigengenes and cell types, with color indicating correlation strength and dot size reflecting signal magnitude. (**F**) Network visualization of hub genes within each module, illustrating co-expression relationships among core and representative genes.

To identify key modules within epithelial cells, we applied hdWGCNA, a dedicated framework for analyzing co-expression networks in scRNA-seq data. First, a scale-free co-expression network was built using an optimal soft-thresholding power of 7 (Figure S5A). This analysis generated a dendrogram that clustered genes into distinct modules based on their expression patterns. Finally, these modules were visualized, each representing a set of co-expressed genes identified through the network evaluation. Every leaf on the dendrogram corresponds to one gene, and the color at the base suggests the allocation of the coexpression module (Figure 3A). The top 21 hub genes based on kME values for each module were visualized (Figure 3B). A total of four distinct gene co-expression modules were identified (Figure 3C,D). The cell type-module correlation analysis demonstrated distinct expression patterns of harmonized module eigengenes across different cellular subsets. Modules showed cell type-specific enrichment, with variations in both expression magnitude (color intensity) and cellular prevalence (dot size) among the identified epithelial cell M1 to M4 (Figure 3E). A module–module correlation matrix highlighted strong positive associations between modules such as PCa-M1 to M3 and M4 to M3, suggesting shared biological functions (Figure S5B). Gene co-expression network topology was visualized through UMAP embedding using the expression patterns of the top 10 hub genes from each module. The spatial arrangement shows distinct clustering of genes by module assignment, with gene point sizes proportional to their intramodular connectivity (kME), illustrating the hierarchical organization of the transcriptional network (Figure 3F). The expression patterns of module eigengenes for each module, colored by their respective module colors, were displayed in the single-cell RNA-seq data (Figure S5C). These findings provide insights into the molecular networks underlying the function of epithelial cells in PCa.

### 3.4. Functional Enrichment Analysis Reveal Key Regulatory Networks in PCa

**Figure 4:**
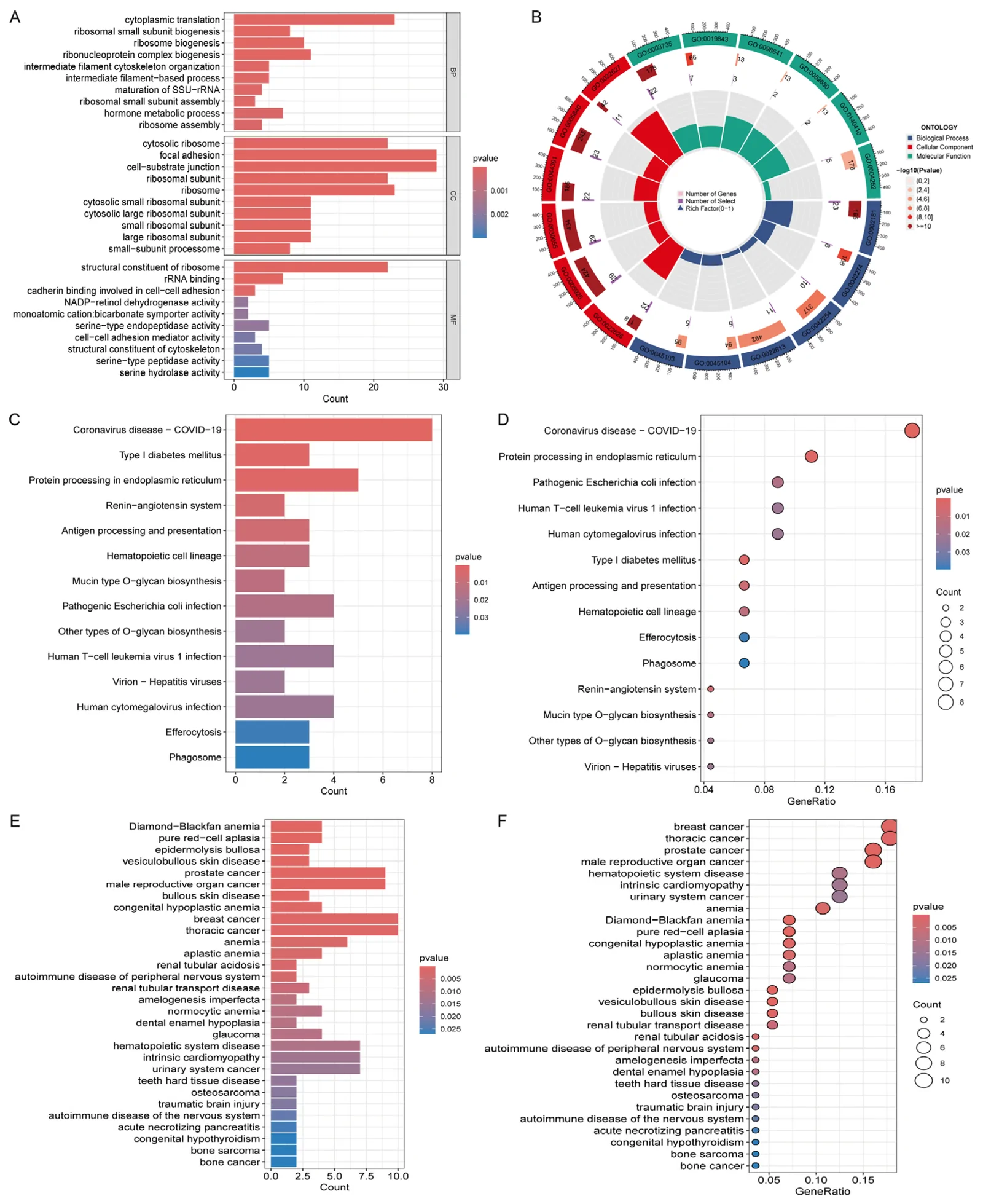
Functional Enrichment Analysis Reveal Key Regulatory Networks in PCa. (**A**) Bar plot of GO enrichment of differentially expressed genes, displaying only the results with adjusted *p* < 0.05. (**B**) Circular plot of GO enrichment of differentially expressed genes, displaying only the results with adjusted *p* < 0.05. (**C**) Bar plot of KEGG enrichment of differentially expressed genes, displaying only the results with *p* < 0.05. (**D**) Bubble plot of KEGG enrichment of differentially expressed genes, displaying only the results with *p* < 0.05. (**E**) Bar plot of DO enrichment of differentially expressed genes, displaying only the results with *p* < 0.05. (**F**) Bubble plot of DO enrichment of differentially expressed genes, displaying only the results with *p* < 0.05.

Functional enrichment analysis of hub DEGs in PCa revealed multilevel regulatory networks through integrated GO and KEGG pathway analyses. GO annotation demonstrated significant enrichment across three functional domains: biological processes showed regulation of peptidase activity and antimicrobial humoral response; cellular component analysis highlighted apical plasma membrane and lysosomal membrane; molecular function assessment identified key roles in peptidase regulator activity and endopeptidase inhibitor activity (Figure 4A,B). KEGG pathway analysis revealed the significantly enriched cancer-related networks of PCa, with the cAMP signaling pathway emerging as the most prominent (Figure 4C,D). Through DO enrichment analysis, we identified hdWGCNA genes term significantly associated with PCa (*p* < 0.05). Among these, PCa terms remained significant after multiple testing correction (*p* < 0.05) (Figure 4E,F). This dysregulation may compromise cellular defense against carcinogens and disrupt epithelial cell-related molecular programs, thereby contributing to PCa progression. These findings provide a comprehensive framework linking molecular alterations to pathway activation and cancer hallmarks in prostate tumorigenesis.

### 3.5. MR of Hub Diagnostic Biomarker

**Figure 5:**
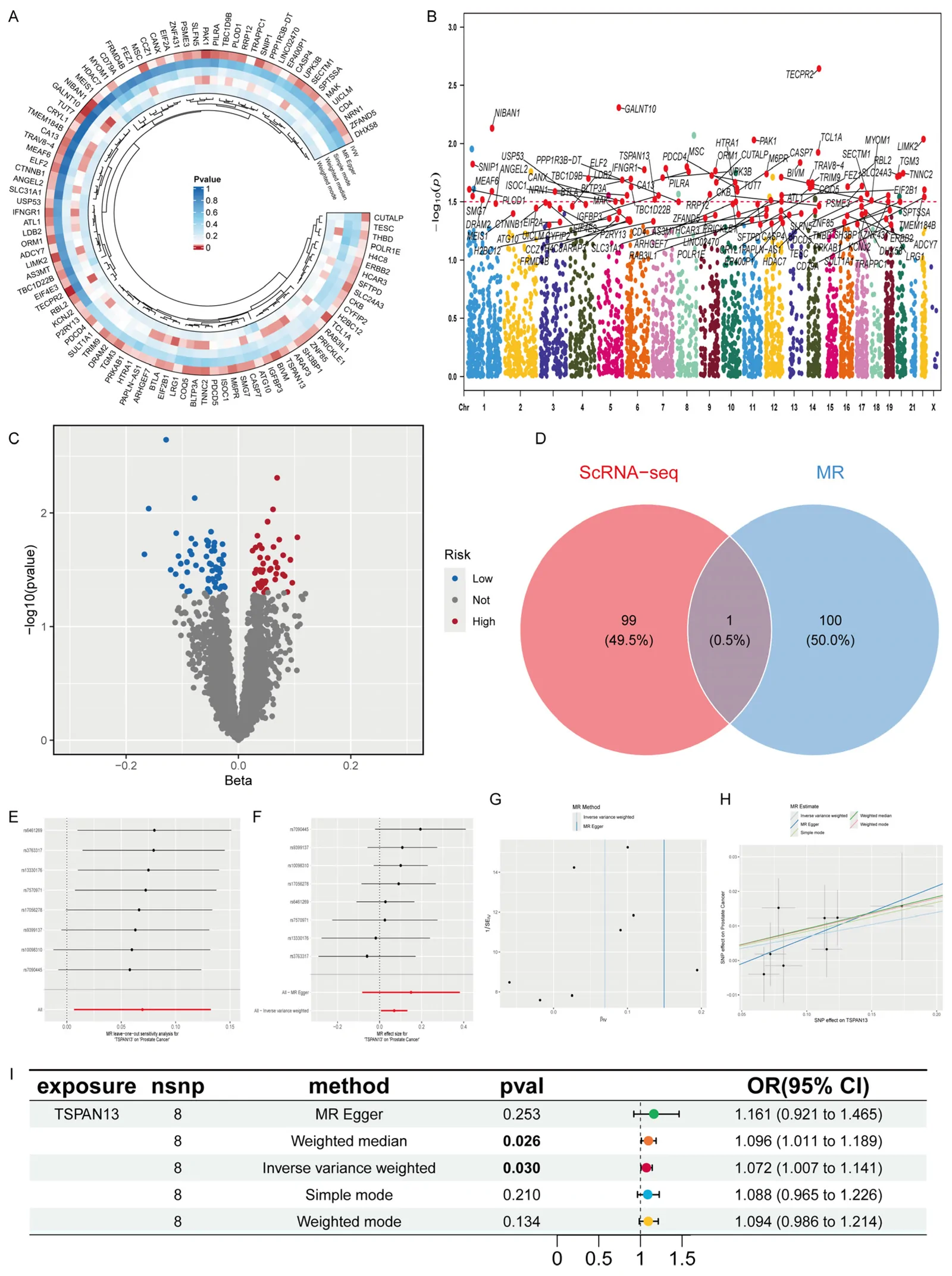
Identification of candidate EMT-associated predictive biomarker for PCa. (**A**) Circular heatmap summarizing Mendelian randomization (MR) results, in which rows represent exposures, columns represent MR methods (including IVW), and colors indicate the corresponding *p*-values. (**B**) Distribution of -log_10_(*p*-values) from Inverse Variance Weighted MR analysis across chromosomal positions. The red horizontal line indicates the significance threshold. Genes surpassing this threshold are highlighted in red and labeled. (**C**) Volcano plot of MR results based on the inverse variance weighted (IVW) method, showing effect sizes (Beta) versus statistical significance −log_10_(*p*-values), with significant genes highlighted and colored by inferred risk direction. (**D**) Venn diagram showing overlapping genes identified as predictive biomarker by ScRNA-seq and MR models. (**E**) Leave-one-out sensitivity analysis evaluating the influence of individual SNPs on the overall MR estimate. (**F**) Forest plot showing single-SNP MR estimates, with effect sizes and 95% confidence intervals for each instrumental variable. (**G**) Funnel plot assessing potential directional pleiotropy, in which symmetry of SNP-specific effects reflects the absence of systematic bias. (**H**) Scatter plot depicting the inverse correlation between TSPAN13 expression levels (*x*-axis) and PCa risk (*y*-axis). (**I**) Forest plot illustrating IVW method identifying TSPAN13 as a significant risk factor against PCa (*p* < 0.05), with the OR and 95% confidence interval shown. The bold indicates significance.

The circular plot analysis, incorporating five distinct MR methods—IVW, MR-Egger regression, weighted median, simple mode, and weighted mode—identified eQTL genes associated with PCa. A smaller *p*-value indicates stronger evidence against the null hypothesis of no association, rather than a greater magnitude of biological effect (Figure 5A). The Manhattan plot illustrates the distribution of *p*-values from Inverse Variance Weighted Mendelian randomization analysis across all autosomes. Each point represents the −log_10_(*p*-value) for a specific gene-exposure association, plotted according to its chromosomal position. Notably, this gene exceeded the significance threshold, with these significant associations being prominently highlighted in red and labeled for identification (Figure 5B). Visualization of MR results via volcano plot revealed distinct patterns of association. The plot displays three distinct groups: red points representing significant risk-increasing factors with positive effect sizes, blue points indicating significant protective factors with negative effect sizes, and gray points corresponding to non-significant associations clustered around the null effect (Figure 5C). Venn diagram intersection analysis (Figure 5D) revealed TSPAN13 as the candidate EMT-associated regulator consistently identified across all computational approaches (MR, ScRNA-Seq). Leave-one-out sensitivity analysis confirmed robustness of the genetic association estimate, with no single SNP disproportionately influencing the overall effect and the scatter plot visualized the positive relationship between hub gene expression and PCa risk across five regression algorithms (Figure 5E). The forest plot (Figure 5F) demonstrated a positive association betweenTSPAN13-related SNPs and PCa risk, indicating TSPAN13 as a risk factor. The funnel plot (Figure 5G) exhibited symmetrical distribution of SNPs, suggesting minimal small-study bias. The scatter plot (Figure 5H) visualized the positive relationship between TSPAN13 expression and PCa risk across five regression algorithms. The upward trend in SNP effect sizes (*x*-axis: hub genes-associated SNPs; *y*-axis: PCa risk) was consistent with a risk-increasing effect. Sensitivity analyses did not detect significant evidence of horizontal pleiotropy (MR-Egger intercept) or outliers (MR-PRESSO), indicating that the candidate EMT-associated regulator instrumental variable assumptions were not grossly violated. This lends greater confidence to the observed genetic association (Table S2). Using IVW analysis as the primary method, we identified a nominal genetic association between genetically predicted TSPAN13 expression and increased prostate cancer risk (OR = 1.072, 95% CI: 1.007–1.141, *p* < 0.05, Figure 5I). This cross-method convergence suggests that TSPAN13 dysregulation may contribute to prostate cancer susceptibility and warrants further investigation as a potential risk-associated biomarker.

### 3.6. Validation of Candidate EMT-Associated Regulator

**Figure 6:**
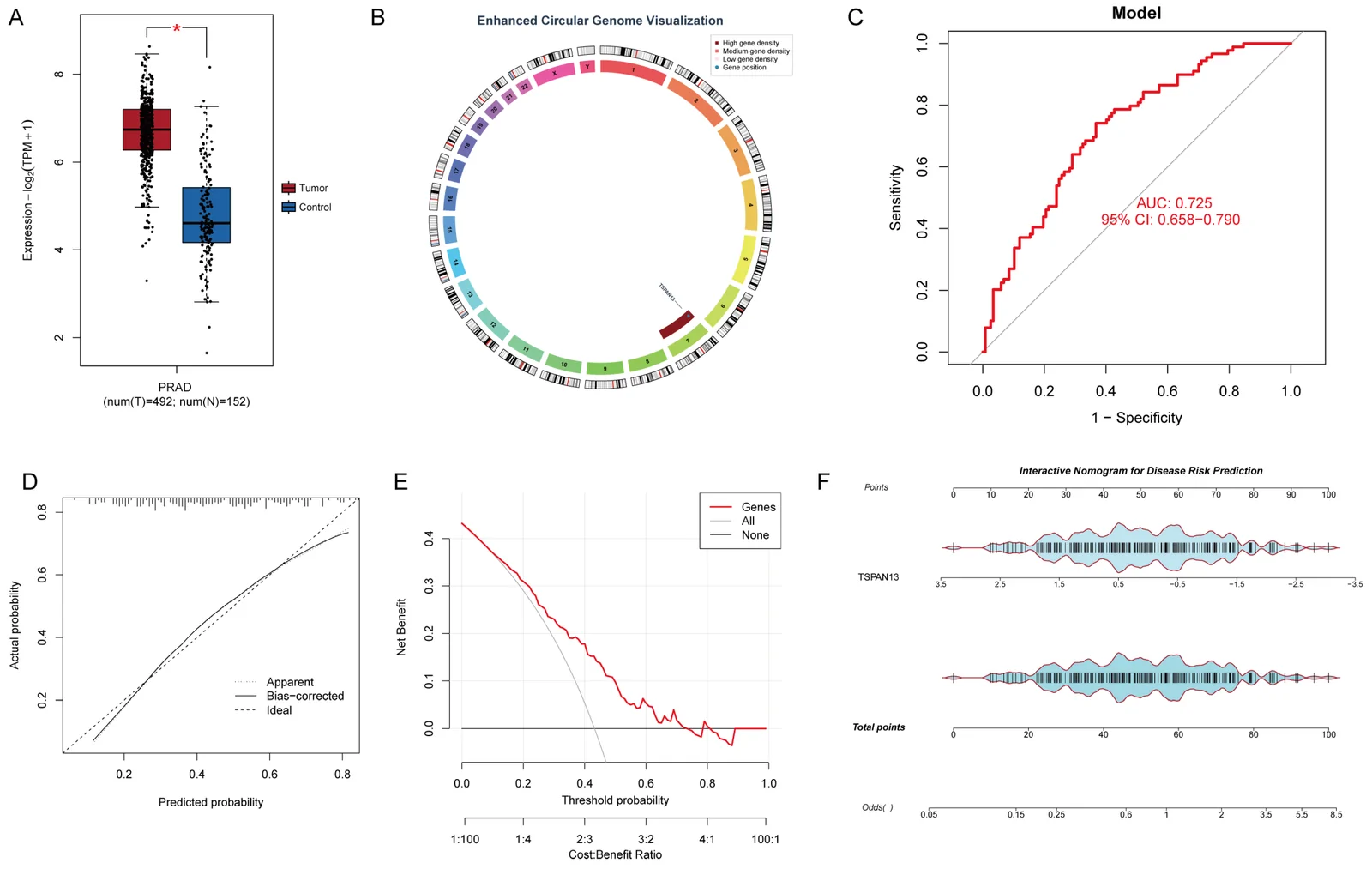
Validation of candidate EMT-associated predictive biomarker. (**A**) Validation of candidate EMT-associated regulator expression in TCGA database, * *p* < 0.05. (**B**) Candidate EMT-associated regulator is visualized via a circular genome plot. (**C**) AUC area plot summarizing diagnostic performance of candidate EMT-associated predictive biomarker. (**D**) Calibration plot evaluating the model’s predictive accuracy. (**E**) Decision Curve Analysis (DCA) plot evaluating potential decision-making utility. (**F**) Nomogram for risk prediction using candidate EMT-associated predictive biomarker.

To validate reproducibility, TCGA datasets confirmed the dysregulated expression signature: TSPAN13 was significantly upregulated (Figure 6A, *p* < 0.05). This gene was subsequently mapped to their respective chromosomal locations, which were found to be distributed in 7th chromosome (Figure 6B). Subsequent ROC curve analysis showed a modest single-marker classification performance, with AUC values of 0.725 for TSPAN13 (95%CI: 0.658–0.790, Figure 6C). Using TCGA-PRAD clinical data, high versus low TSPAN13 expression was not significantly associated with overall survival (HR = 0.82, *p* = 0.75; Figure S6). Calibration plots showed apparent agreement between predicted and observed outcomes (Figure 6D). Decision curve analysis suggested a potential net benefit across a range of threshold probabilities in this dataset (Figure 6E). Integrating feature importances, we developed an exploratory diagnostic nomogram incorporating TSPAN13 (Figure 6F). Collectively, these findings suggest that TSPAN13 may serve as a candidate diagnostic biomarker for PCa, warranting further validation.

### 3.7. Immune Microenvironment Characterization in PCa

**Figure 7:**
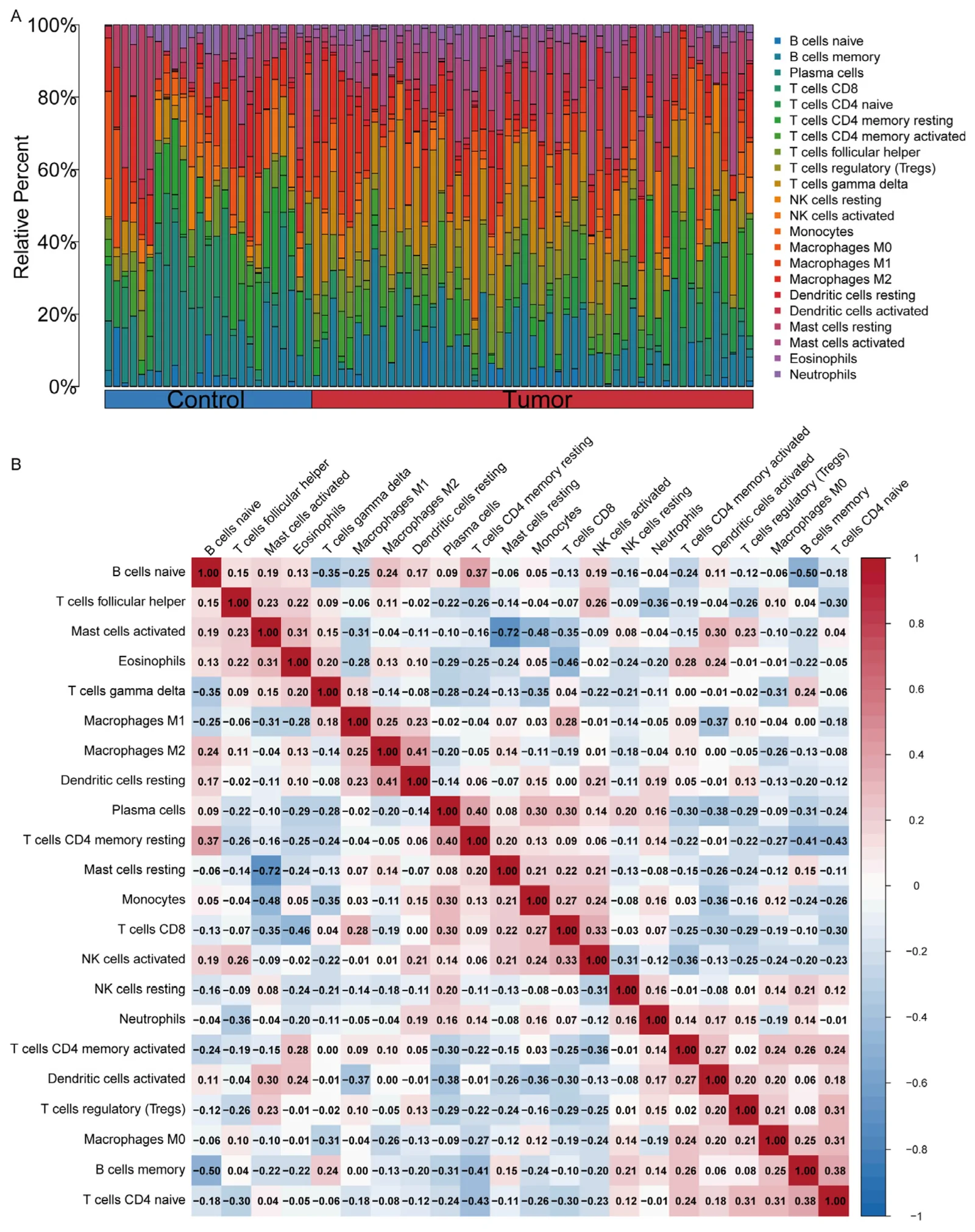
Immune cell infiltration analysis of CIBERSORT. (**A**) Bar plots of immune cell composition proportions. (**B**) Correlation heatmap of immune cell interactions.

**Figure 8:**
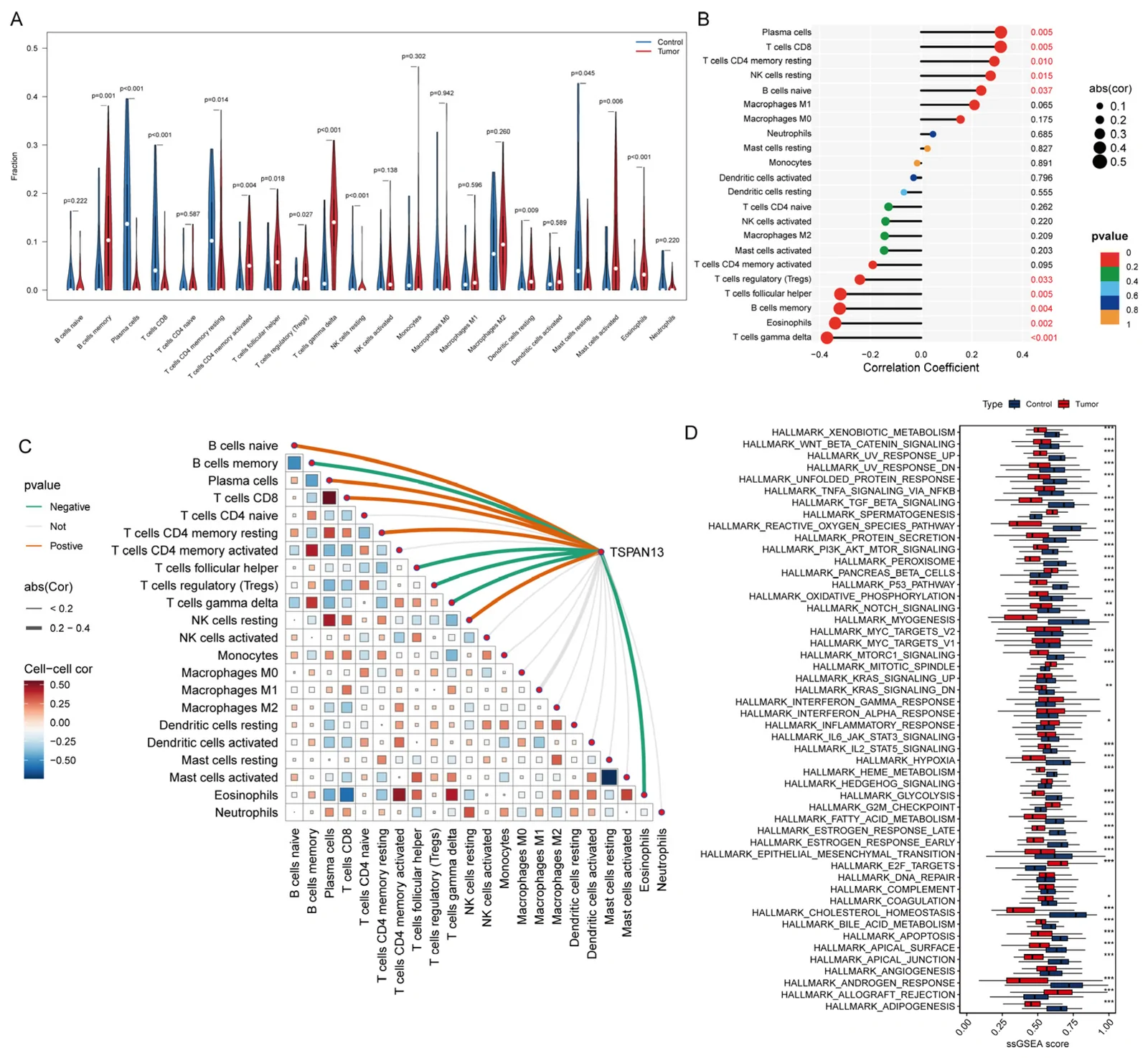
Characterization of tumor immune microenvironment and drug enrichment analysis. (**A**) Violin plots comparing immune cell infiltration scores between Tumor and Control groups, with statistical significance (*p* < 0.05). (**B**) Lollipop plot showing Spearman’s rank correlation coefficients between TSPAN13 and immune cell types. (**C**) LinkET plot visualizing the correlation network between TSPAN13 expression levels and immune cell subsets, where node size represents correlation strength and color indicates directionality. (**D**) ssGSEA reveals Hallmark pathways significantly altered in control and tumor, * *p* < 0.05, ** *p* < 0.01, *** *p* < 0.001.

An initial assessment of immune microenvironment differences between control and tumor groups was performed using the CIBERSORT algorithm. To further validate and quantify these findings, the same algorithm was applied to estimate the relative abundance of 22 immune cell types (Figure 7A). Furthermore, Spearman correlation analysis uncovered significant interconnections among most immune cell populations, pointing to complex regulatory dynamics in the tumor microenvironment (Figure 7B). Specifically, B cells memory, T cells regulatory and NK activated cells were significantly increased in tumor samples compared to controls. Conversely, decreased infiltration was observed for plasma cells, T cells CD4 memory resting, and NK resting cells in PCa tissues (*p* < 0.05; Figure 8A). Strikingly, correlation analysis between immune cells and TSPAN13 (Figure 8B). LinkET plot visualizing correlations between TSPAN13 and immune cells (Figure 8C). Scatter plots were generated for immune cells with significant correlations (Figure S7). The ssGSEA analysis revealed significant alterations in Hallmark pathway activities between control and tumor groups (Figure 8D). The identified immunophenotypic associations position TSPAN13 as associated with immune infiltration, suggesting potential mechanistic insights into its tumor-modulatory functions. Collectively, these results suggest that TSPAN13 may act as an EMT-associated regulator potentially involved in immune microenvironment modulation in PCa, warranting further mechanistic and functional validation.

### 3.8. Drug Enrichment Analysis and Molecular Docking

**Figure 9:**
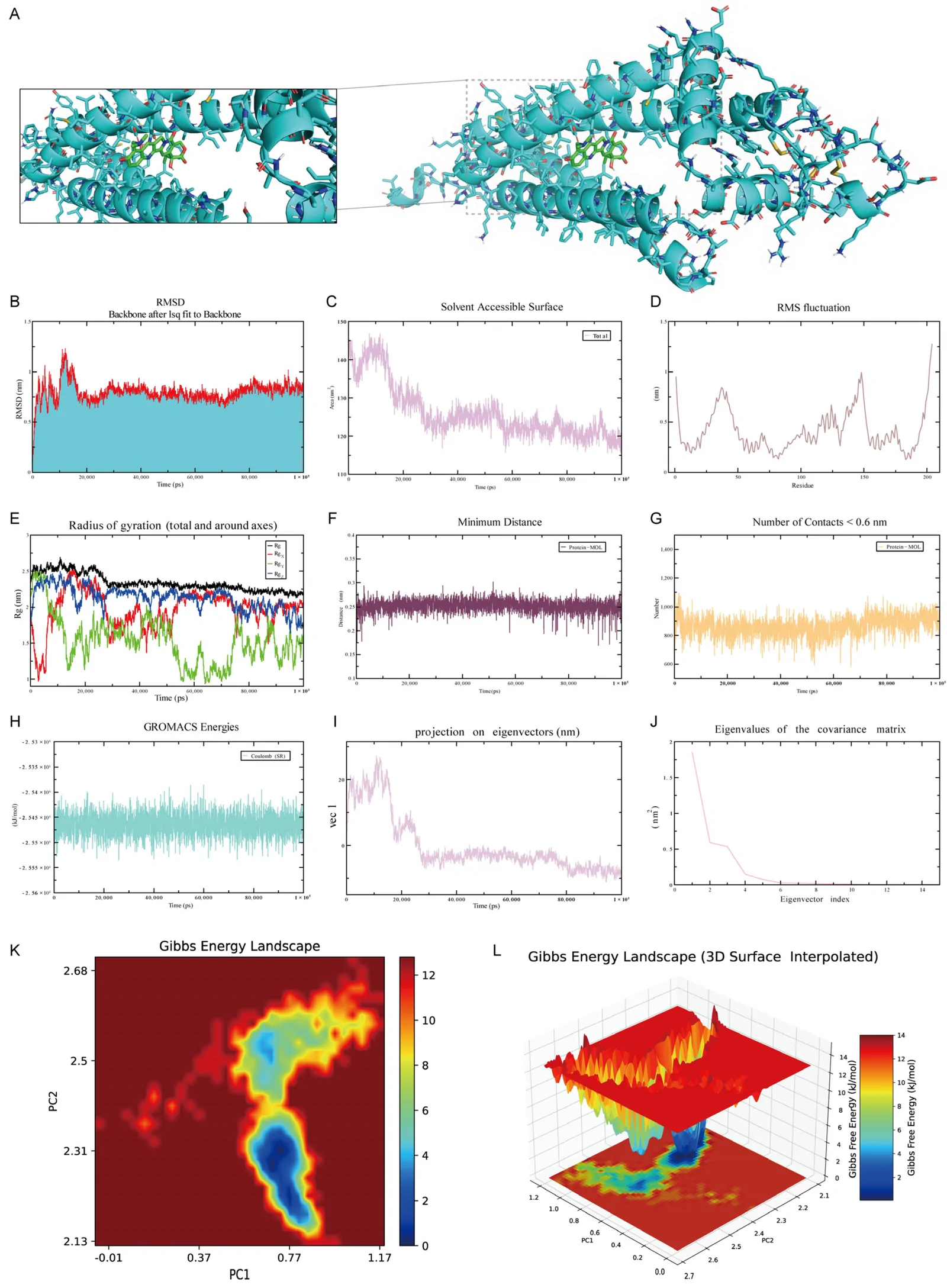
Molecular docking and dynamics simulation analysis of the topotecan-TSPAN13 complex. (**A**) Predicted binding pose of topotecan within the TSPAN13 protein. (**B**) Root-mean-square deviation of the protein–ligand complex backbone. (**C**) Solvent-accessible surface area (SASA) of the complex. (**D**) Root-mean-square fluctuation (RMSF) per residue for the TSPAN13 protein. (**E**) Time evolution of the total radius of gyration (Rg) and its spatial components (Rgx, Rgy, Rgz). (**F**) Minimum distance between the topotecan ligand and the TSPAN13 protein over time. (**G**) Number of atomic contacts (distance < 0.6 nm) between the protein and ligand. (**H**) Short-range Coulombic interaction energy between topotecan and TSPAN13. (**I**) Projection of the MD trajectory onto the first principal component (PC1). (**J**) Magnitude (square root of eigenvalues) of the first 15 principal components (PCs) from PCA. (**K**) Two-dimensional Gibbs free energy landscape projected onto the first two principal components (PC1 and PC2). (**L**) Three-dimensional representation of the Gibbs free energy landscape.

To identify potential therapeutic agents targeting the candidate EMT-associated regulator TSPAN13, we performed a drug enrichment analysis using the DSigDB database. This analysis identified topotecan, a nuclear topoisomerase I inhibitor, as significantly enriched for TSPAN13 (adjusted *p* < 0.05), suggesting a potential therapeutic interaction. Molecular docking was then employed to predict the binding mode of topotecan to TSPAN13, with key protein–ligand interactions visualized (Figure 9A). To further explore the computationally predicted binding mode, we conducted a 100 ns molecular dynamics simulation of the topotecan-TSPAN13 complex. The system stability was assessed through multiple parameters. The root-mean-square deviation (RMSD) of the complex stabilized around 0.75 nm after 20 ns, exhibiting only minor fluctuations, which indicates a stable binding conformation was achieved (Figure 9B). The solvent-accessible surface area (SAS) remained consistently near 120 nm^2^ throughout the simulation, reflecting stable protein–solvent interactions and no major structural rearrangements (Figure 9C). Analysis of residue flexibility via root-mean-square fluctuation (RMSF, 0–1.5 nm) revealed distinct dynamic regions. High fluctuations in residues 25–50 and 125–150 suggest local flexibility, potentially associated with functional or binding sites. Conversely, low fluctuations in regions 75–100 and 175–200 indicate stable, rigid structural domains (Figure 9D). The radius of gyration (Rg) remained constant at approximately 2.25 nm, confirming the overall structural compactness was maintained. Comparable Rg components (RGx, RGy, RGz) suggest similar expansion along all three spatial axes (Figure 9E). Direct metrics of the binding interface suggested a persistent interaction. The minimum protein–ligand distance averaged 0.25 nm (Figure 9F), and approximately 900 atomic contacts were maintained within a 0.6 nm cutoff (Figure 9G). The calculated total protein–ligand interaction energy was favorable, ranging from −2.55 × 10^6^ to −2.545 × 10^6^ kJ/mol (Figure 9H). Principal component analysis (PCA) provided insights into collective motions. Projection along the first principal component (PC1) showed oscillatory motion (−10 to 0 nm), consistent with breathing dynamics of the binding pocket (Figure 9I). The eigenvalue spectrum showed a sharp decay for PC1–PC5, followed by a plateau for higher-order components (PC6–PC15), indicating that the dominant large-scale motions are described by the first few eigenvectors. The largest eigenvector (~1.8 nm) corresponds to the most significant global motion (Figure 9J). Finally, the Gibbs free energy landscape projected onto PC1 and PC2 revealed a single, deep global minimum, characterizing the dominant stable conformation of the complex (Figure 9K). A 3D representation of this landscape further highlighted domains with highly correlated dynamics, aligning with key functional regions of the protein (Figure 9L). Collectively, these computational results—from initial enrichment to detailed dynamic characterization—provide computational evidence supporting topotecan as a computationally predicted candidate.

### 3.9. TSPAN13 Knockdown Attenuates PCa Cell Line Proliferation, Metastasis and EMT-Marker Changes

**Figure 10:**
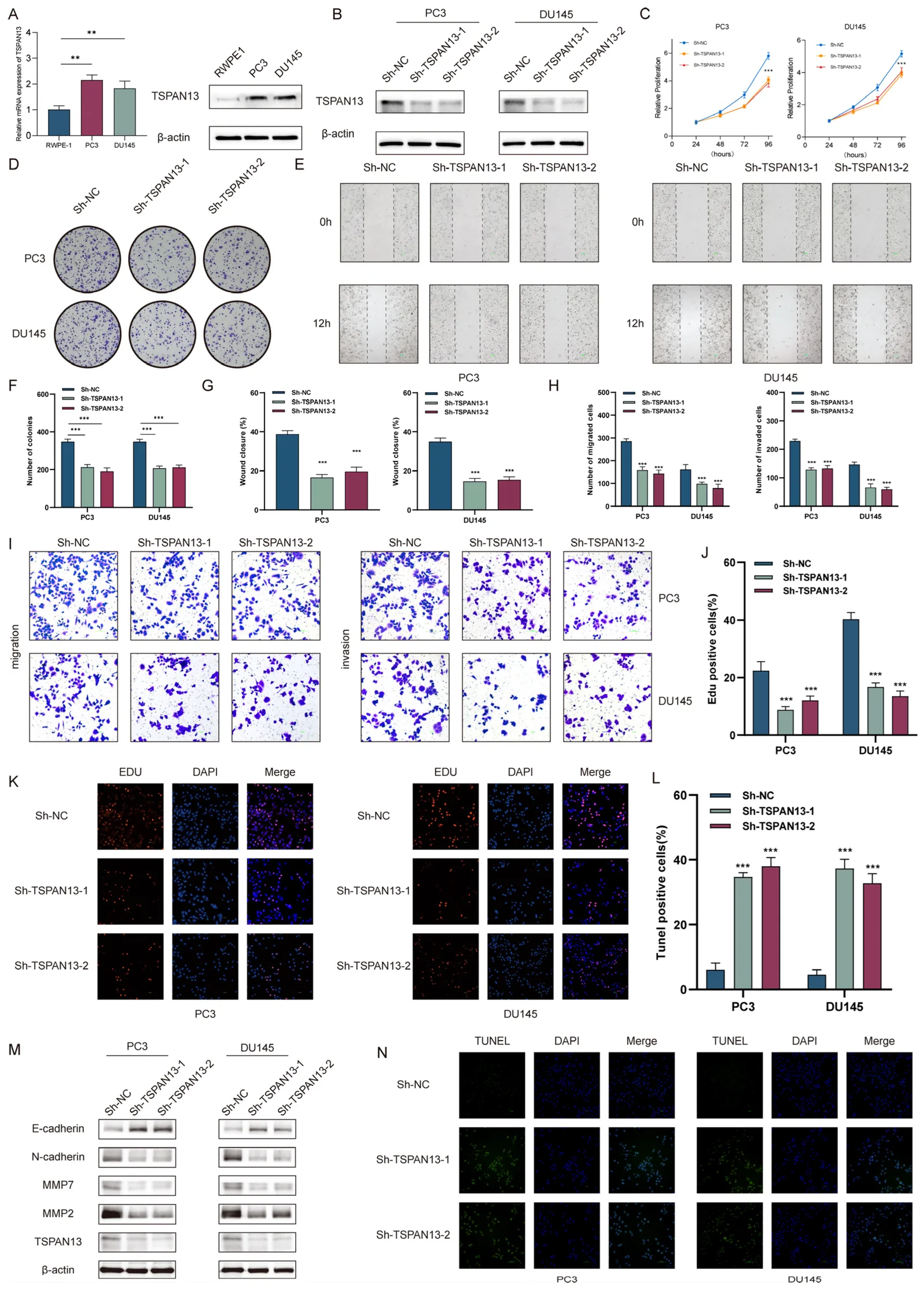
The Role of TSPAN13 in Driving Proliferation, Metastasis, and EMT in Prostate Cancer Cells. (**A**,**B**) qRT-PCR and Western blot analyses confirming efficient knockdown of TSPAN13 by shRNA in PCa cell lines. Data are representative of three independent biological replicates (*n* = 3). (**C**) CCK-8 assays showing that TSPAN13 silencing significantly inhibits PCa cell proliferation (*p* < 0.05). (**D**,**F**) Colony formation assays demonstrating reduced colony number upon TSPAN13 knockdown. (**E**,**G**) Wound healing assays indicating reduced cell migration after TSPAN13 suppression, scale bar: 100 µm. (**H**,**I**) Transwell assays revealing decreased invasive and migratory cell counts in TSPAN13-depleted groups, scale bar: 100 µm. (**J**,**K**) EDU incorporation assays evaluating the proliferative capacity of TSPAN13-knockdown PCa cells, scale bar: 100 µm. (**L**,**N**) TUNEL assays examining apoptosis in PCa cells following TSPAN13 deletion, scale bar: 100 µm. (**M**) Western blot analysis of EMT-related marker expression in TSPAN13-silenced cells. ** *p* < 0.01, *** *p* < 0.001.

To experimentally define the contribution of TSPAN13 to PCa malignancy, we performed a series of in vitro functional assays. We first assessed its endogenous expression levels in two widely used PCa cell lines, PC3 and DU145. Both WB analysis and qRT-PCR consistently showed that TSPAN13 expression was significantly upregulated in these PCa cell lines compared to the normal prostate epithelial cell line RWPE-1 (Figure 10A). To silence TSPAN13 via RNA interference, we employed two specific shRNAs (sh-TSPAN13-1 and sh-TSPAN13-2), with a non-targeting shRNA (shNC) as a control. WB analysis confirmed effective knockdown of TSPAN13 by both targeting shRNAs (Figure 10B). Loss of TSPAN13 expression induced an anti-tumorigenic phenotype, significantly suppressing both cell proliferation (Figure 10C) and colony formation (Figure 10D,F) capacity compared to the shNC control. In scratch wound healing assays, TSPAN13-deficient cells exhibited markedly reduced migration at 12 h relative to controls (Figure 10E,G). Transwell assays further demonstrated that TSPAN13 knockdown concurrently decreased both migration and invasion, with fewer cells migrating or invading in the sh-TSPAN13-1 and sh-TSPAN13-2 groups compared to the control (Figure 10I,H). Consistent with the observed suppression of proliferation, EdU incorporation assays revealed a significant reduction in the percentage of cells actively synthesizing DNA following TSPAN13 knockdown. The proportion of EdU-positive cells was markedly lower in both the sh-TSPAN13-1 and sh-TSPAN13-2 groups compared to the shNC control (Figure 10K,J). In contrast, the apoptosis rate was markedly elevated in both cell types following TSPAN13 inhibition (Figure 10N,L). Notably, TSPAN13 silencing also affected key molecular markers of EMT. Specifically, the expression of the epithelial marker E-cadherin was increased, whereas levels of the mesenchymal marker N-cadherin, as well as the matrix metalloproteinases MMP2 and MMP7, were suppressed upon TSPAN13 knockdown (Figure 10M and Figure S8). These changes suggest that TSPAN13 may contribute to an EMT-like phenotype in PCa cells. Together, our findings indicate that TSPAN13 knockdown attenuates proliferation, migration, invasion, and EMT-marker changes in the tested PCa cell models.

### 3.10. TSPAN13 Knockdown Suppresses PCa Growth In Vivo and Induces Regulatory Network Perturbations

**Figure 11:**
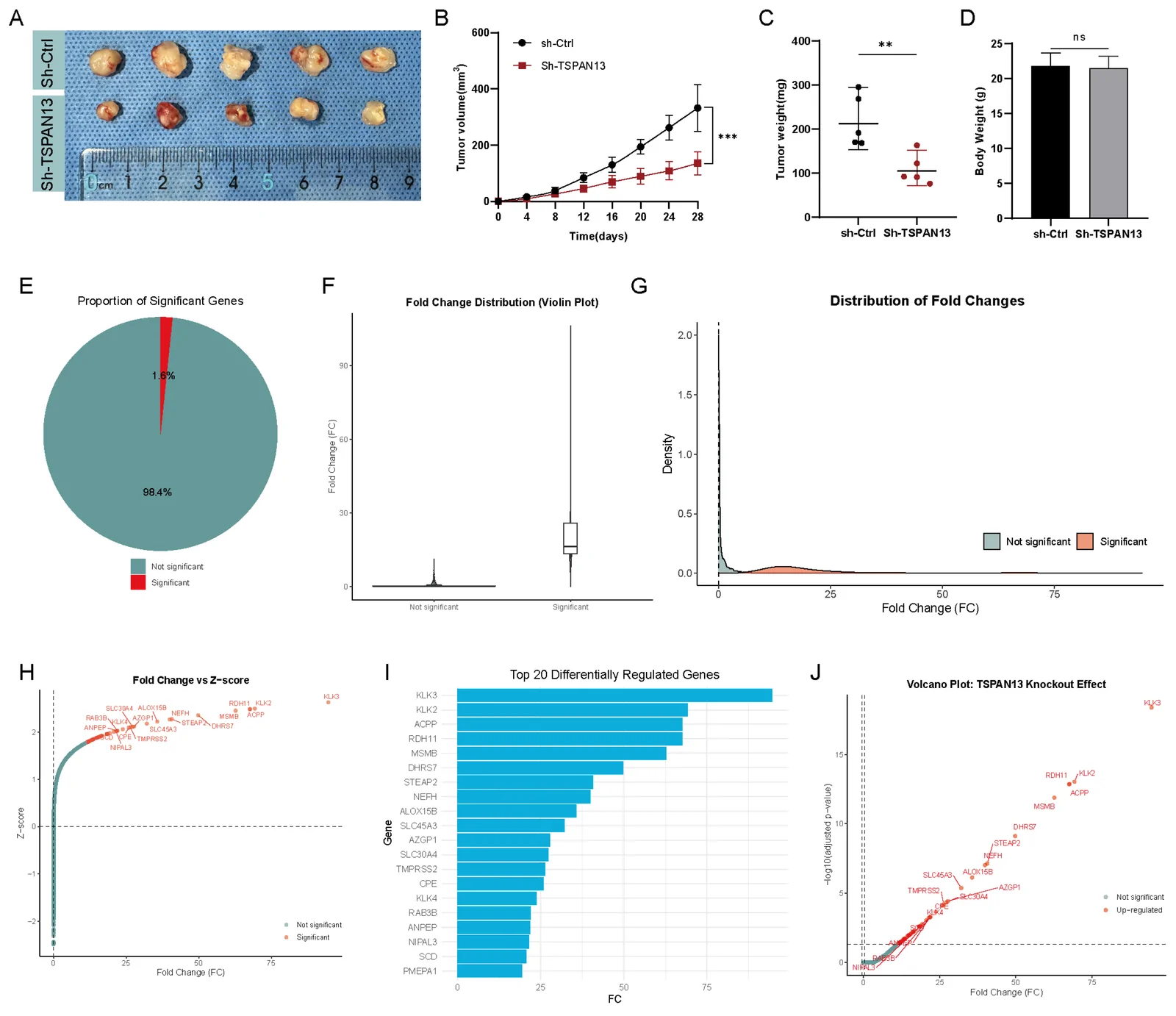
TSPAN13 knockdown suppresses prostate cancer growth in vivo and induces regulatory network perturbations. (**A**) Xenograft tumors resected from nude mice. Mice were implanted with prostate cancer cells expressing either control shRNA and shTSPAN13. (**B**) Tumor growth curves showing tumor volume progression in shNC and shTSPAN13 groups. (**C**) Final tumor weights at the experimental endpoint. (**D**) Body weight monitoring of mice during the course of the experiment, showing no significant change in body weight between groups. (**E**) Proportion of genes exhibiting significant changes in network-derived regulation scores following in silico knockout of TSPAN13, as determined by scTenifoldKnk analysis. (**F**,**G**) Distribution of fold changes derived from network perturbation analysis, illustrating the magnitude of regulatory alterations induced by TSPAN13 perturbation. (**H**) Scatter plot of fold change versus Z score highlighting genes with strong and statistically significant regulatory perturbations following in silico knockout of TSPAN13. (**I**) Bar plot displaying the top 20 genes exhibiting the most pronounced regulatory perturbations. (**J**) Volcano plot visualizing genes sensitive to perturbation of the TSPAN13-centered regulatory network, with KLK2 and KLK3 highlighted. ** *p* < 0.01, *** *p* < 0.001, ns indicates not significant.

To evaluate the tumor-suppressive function of TSPAN13 in vivo, control (shNC) or TSPAN13-knockdown (shTSPAN13) cells were subcutaneously injected into male nude mice to establish xenograft tumor models (Figure 11A). Consistent with the in vitro findings, tumors derived from shTSPAN13 cells exhibited significantly reduced tumor volumes and lower final tumor weights compared with the shNC group (Figure 11B,C). No significant difference in total body weight was observed between the two groups throughout the experiment, indicating that the inhibitory effect on tumor growth was not attributable to systemic toxicity (Figure 11D). To further investigate the global regulatory consequences associated with TSPAN13 loss, an in silico knockdown analysis was performed based on single-cell RNA sequencing data. This analysis revealed that approximately 1.6% of genes exhibited significant changes in network-derived regulation scores following perturbation of the TSPAN13-centered regulatory network (Figure 11E). Among these genes, the median fold change, reflecting the magnitude of regulatory perturbation rather than transcriptional expression change, was 15 (Figure 11F,G). A scatter plot of fold change versus Z score was used to visualize the regulatory perturbations induced by in silico knockout of TSPAN13. Genes with high absolute fold change and Z score represent those exhibiting strong and statistically significant alterations in network-derived regulatory states (Figure 11H). A bar plot displaying the top 20 genes with the most pronounced regulatory perturbations is shown in Figure 11I. Notably, KLK3 and KLK2 were highlighted among the genes most sensitive to perturbation of the TSPAN13-associated regulatory network in the volcano plot (Figure 11J).

## 4. Discussion

In this study, through an integrative multi-omics approach encompassing single-cell sequencing, Mendelian randomization, and machine learning, we identified and validated TSPAN13 as a key gene significantly upregulated in PCa epithelial cells. This finding positions TSPAN13 as a potential contributor to PCa pathogenesis. KEGG pathway analysis identified cAMP signaling as the most significantly enriched pathway among the hub-gene modules. This is consistent with the known role of cAMP signaling in prostate epithelial cell proliferation, differentiation, and androgen-independent survival. However, whether TSPAN13 directly modulates cAMP signaling was not tested in this study, and this finding should therefore be considered hypothesis-generating and requires further mechanistic validation. Parallel analysis revealed a profound remodeling of the tumor immune microenvironment in PCa, characterized by increased infiltration of memory B cells, regulatory T cells (Tregs), and activated NK cells, alongside a decrease in plasma cells, resting CD4^+^ memory T cells, and resting NK cells. This specific immune contexture suggests a suppressive microenvironment that may facilitate tumor evasion.

Intriguingly, preliminary analyses suggest a potential correlation between TSPAN13 expression and sensitivity to topoisomerase I inhibitors, such as Topotecan (TPT). TPT is a semi-synthetic derivative of camptothecin, a pentacyclic alkaloid originally isolated from the *Camptotheca acuminata* tree. Its antitumor activity is mediated through two primary mechanisms: first, by inhibiting the nuclear enzyme topoisomerase I, it induces lethal double-strand DNA breaks during the S-phase of replication; second, it downregulates the activity of hypoxia-inducible factors. This agent has received clinical approval for the treatment of several malignancies, including cervical cancer, small-cell lung cancer, medulloblastoma, neuroblastoma, rhabdomyosarcoma and recurrent ovarian cancer [30,31,32,33,34,35]. To explore the potential interaction between TPT and TSPAN13, we performed computational analyses. Molecular docking and long-timescale molecular dynamics simulations suggested that TPT may stably associate with TSPAN13, forming persistent hydrogen-bonding and hydrophobic contacts and occupying energetically favorable conformations. These results indicate a computationally predicted binding mode, providing a hypothesis that TSPAN13 may be a potential binding partner of TPT in PCa cells; however, this remains to be experimentally validated. By contrast, TSPAN13 was identified as a tumor suppressor [36]; Huang et al. demonstrated growth- and invasion-suppressive effects of ectopic NET-6/TSPAN13 in a breast cancer model [37]. Jiang et al. reported associations with less aggressive breast tumor features [15]. The prostate study by Arencibia et al. reported increased TSPAN13 expression in cancer, together with an inverse association with Gleason score [38], whereas our study suggests a tumor-promoting role in the PC3 and DU145 models. We propose that this discrepancy may be attributed to the following factors: (1) differences in the histological and molecular heterogeneity of prostate cancer patient cohorts; (2) variations in sample size, data processing pipelines, and clinical annotation standards across datasets; and (3) the possibility that TSPAN13 exerts distinct functions depending on the tumor microenvironment and genetic background. Our multi-omics analysis and in vitro/in vivo functional assays support a candidate EMT-associated tumor-promoting role for TSPAN13 in these models.

The elevated expression of TSPAN13 in epithelial cells prompts an intriguing hypothesis regarding its functional role. In the field of tumor biology, the EMT is a critical cellular process. This process is characterized by the downregulation of epithelial features and the acquisition of mesenchymal phenotypes and behaviors [39,40]. During tumor invasion, EMT plays a central role: epithelial cells lose their polarity and cell–cell contacts, undergo profound cytoskeletal remodeling, and thereby gain migratory and invasive capabilities [41,42,43]. Furthermore, EMT is recognized as a key driver of metastatic dissemination and a major contributor to cancer-related mortality [44]. By acquiring a mesenchymal phenotype through EMT, tumor cells gain enhanced migratory and invasive properties, thereby promoting metastasis from the primary site. Given the established importance of EMT in tumor invasion and metastasis, we speculate that TSPAN13 may participate in regulating this process. Its high expression in cancerous epithelial cells could potentially drive the loss of epithelial features and enhance migratory capabilities, thereby contributing to local invasion and the seeding of distant metastases. Furthermore, the observed immunosuppressive landscape, particularly the enrichment of Tregs, might create a permissive niche for EMT and cancer cell dissemination. To test this, we established a stable TSPAN13-knockdown model in a PCa cell line. Experimental validation confirmed that TSPAN13 depletion significantly attenuated malignant phenotypes: it suppressed cellular proliferation and colony formation, reduced migratory and invasive capacities in transwell assays, and altered the expression of key EMT markers as shown by Western blot. Moreover, TSPAN13 knockdown exerted a tumor-suppressive effect in vivo, as evidenced by significantly reduced tumor growth in xenograft models, consistent with a tumor-promoting role for TSPAN13 in these models. Importantly, the absence of changes in body weight suggests that the observed tumor inhibition is not attributable to general toxicity, supporting a specific role for TSPAN13 in regulating tumor growth. These functional data suggest that TSPAN13 may contribute to an EMT-like phenotype and support its association with proliferative and invasive behaviors in PCa, consistent with our computational predictions and highlighting its role as a critical node in PCa progression. To further explore the molecular consequences associated with loss of TSPAN13, we employed a network-based in silico knockout approach using scTenifoldKnk. Unlike conventional differential expression analyses, this framework assesses changes in gene regulatory states inferred from single-cell transcriptomic data, enabling the identification of genes that are particularly sensitive to perturbation of a target-centered regulatory network. Our analysis revealed that disruption of TSPAN13 is associated with widespread regulatory network rewiring, affecting a distinct subset of genes rather than inducing global transcriptional alterations.

While our study provides novel insights into the potential regulatory role of TSPAN13 in PCa progression through integrated bioinformatics analysis and functional validation. However, several aspects warrant further investigation: First, while our analysis primarily leveraged publicly available transcriptomic datasets to identify TSPAN13-related patterns, the relatively limited sample size in these cohorts necessitates cautious interpretation of the observed associations. Future studies should incorporate prospectively collected multi-institutional cohorts with standardized processing protocols to validate the observed TSPAN13 expression patterns and their clinical correlations. Second, our functional experiments were performed only in PC3 and DU145 cells, both of which are AR-negative/AR-indifferent prostate cancer models. Although these cell lines are widely used to study prostate cancer, they do not represent AR-positive prostate cancer. Therefore, the EMT-associated tumor-promoting role of TSPAN13 observed in our study may be specific to AR-negative/AR-independent prostate cancer and should not be directly extrapolated to AR-positive disease. In addition, our functional data were based on shRNA knockdown without rescue or gain-of-function validation, limiting causal inference and not fully excluding off-target effects. Third, the mechanistic insights presented here are currently derived from cell culture models with restricted biological replicates; clinical tissue microarray analyses with expanded sample size, spatial validation and immune profiling are essential to confirm the translational relevance of our cellular findings. Fourth, we did not assay canonical EMT transcription factors, upstream EMT-related signaling pathways, or TSPAN13 interaction partners. Therefore, the precise molecular mechanisms by which TSPAN13 regulates EMT and the immune microenvironment remain to be fully elucidated. Collectively, these limitations emphasize that our current work represents a foundational step rather than a conclusive statement. Addressing these gaps through integrated multi-approaches, standardized preclinical modeling, and rigorously designed clinical studies will be essential to fully elucidate TSPAN13’s role in PCa biology and its therapeutic potential.

## 5. Conclusions

In summary, this study identifies TSPAN13 as an EMT-associated regulator in PCa. Using a multi-modal approach combining transcriptomics, computational simulation, and functional assays, we demonstrate that TSPAN13 promotes PCa progression by maintaining an EMT-conducive state, enhancing proliferative and invasive capacities. Our findings not only deepen the understanding of PCa biology but also provide a basis for further investigation of TSPAN13 as a candidate therapeutic target, including its computationally predicted potential for combination with agents such as topoisomerase inhibitors.

## Data Availability

The data presented in this study are available in Gene Expression Omnibus at https://www.ncbi.nlm.nih.gov/geo/, accessed on 2 August 2025, reference number GSE176031, GSE60329, GSE71781.

## Supplementary Materials

The following supporting information can be downloaded at https://www.mdpi.com/article/10.3390/biomedicines14102241/s1. Figure S1: Standardized preprocessing procedures of raw data. (A) Box diagrams of GSE60329 and GSE71781 before calibration. (B) Box diagrams of GSE60329 and GSE71781 after calibration. (C) Principal component analysis of GSE60329 and GSE71781 before calibration. (D) Principal component analysis of GSE60329 and GSE71781 after calibration. Figure S2: Workflow for Single-Cell RNA-Seq Data Quality Control and Preprocessing. (A) Visualization of key QC metrics: total RNA counts (nCount_RNA), number of detected genes (nFeature_RNA), and mitochondrial gene proportion. (B) Scatter plots showing the relationship between mitochondrial content and sequencing depth/nFeature_RNA. (C) Identification of the top 1500 highly variable genes. (D) Elbow plot used to determine the number of significant principal components. (E) Principal component analysis (PCA) results. (F) JackStraw plot for statistical validation of selected principal components (PC1-PC20). (G) Cell trajectory analysis (pseudotime) depicting the inferred progression of tumor cells. (H) Pseudotime distribution of tumor cells across the 7 identified cell types. Figure S3: Heatmap displaying the differential expression of the top 10 marker genes across the 18 identified clusters. Figure S4: Left panel: Dynamic patterns of representative DEGs for each cell population. Middle panel: Heatmap showing the representative DEGs across each cell population. Right panel: Representative enriched gene ontology (GO) terms for each cell population. Figure S5: Single-cell hdWGCNA reveals co-expression modules and hub genes in epithelial cells. (A) Soft-thresholding analysis showing scale-free topology fit and mean connectivity across candidate powers, used to select the optimal soft power (soft power = 7). (B) Module–module correlation heatmap depicting the relationships among identified co-expression modules. (C) UMAP of genes colored by module assignment and scaled by kME, revealing the spatial organization of module genes. Figure S6: Kaplan–Meier analyses of TSPAN13 expression in TCGA-PRAD. High versus low TSPAN13 expression was not significantly associated with overall survival. Figure S7: Correlation plot visualizing correlations between TSPAN13 and immune cells. Figure S8: Densitometric quantification of EMT-related protein expression shown in Figure 10M. Western blot bands for E-cadherin, N-cadherin, MMP2, and MMP7 were quantified by densitometry and normalized to β-actin. PC3 and DU145 cells were transfected with sh-NC, sh-TSPAN13-1, or sh-TSPAN13-2. Table S1. Predicted molecular interactions between TSPAN13 and its putative binding partners. Table S2. Sensitivity analyses for Mendelian randomization: heterogeneity Q statistics (IVW and MR-Egger) and MR-Egger intercept test for pleiotropy.

## Abbreviations

The following abbreviations are used in this manuscript:

PCa	Prostate Cancer
EMT	Epithelial–mesenchymal Transition
GEO	Gene Expression Omnibus
hdWGCNA	High-dimensional Weighted Gene Co-expression Network Analysis
GO	Gene Ontology
KEGG	Kyoto Encyclopedia of Genes and Genomes
DO	Disease Ontology
MR	Mendelian Randomization
SNPs	Single-Nucleotide Polymorphisms
IVs	Instrumental Variables
IVW	Inverse Variance Weighted
MD	Molecular Dynamics
